# The interaction between ageing and Alzheimer's disease: insights from the hallmarks of ageing

**DOI:** 10.1186/s40035-024-00397-x

**Published:** 2024-01-23

**Authors:** Yuqing Liu, Yejun Tan, Zheyu Zhang, Min Yi, Lemei Zhu, Weijun Peng

**Affiliations:** 1 Department of Integrated Traditional Chinese and Western Medicine, The Second Xiangya Hospital, Central South University, No.139 Middle Renmin Road, Changsha, 410011 Hunan People’s Republic of China; 2 National Clinical Research Center for Metabolic Diseases, Changsha, 410011 People’s Republic of China; 3 School of Mathematics, University of Minnesota Twin Cities, Minneapolis, MN 55455 USA; 4 Academician Workstation, Changsha Medical University, Changsha, 410219 People’s Republic of China

**Keywords:** Alzheimer’s disease, Brain ageing, Hallmarks of ageing, Cell senescence, Chronic inflammation, Anti-ageing therapy

## Abstract

Ageing is a crucial risk factor for Alzheimer’s disease (AD) and is characterised by systemic changes in both intracellular and extracellular microenvironments that affect the entire body instead of a single organ. Understanding the specific mechanisms underlying the role of ageing in disease development can facilitate the treatment of ageing-related diseases, such as AD. Signs of brain ageing have been observed in both AD patients and animal models. Alleviating the pathological changes caused by brain ageing can dramatically ameliorate the amyloid beta- and tau-induced neuropathological and memory impairments, indicating that ageing plays a crucial role in the pathophysiological process of AD. In this review, we summarize the impact of several age-related factors on AD and propose that preventing pathological changes caused by brain ageing is a promising strategy for improving cognitive health.

## Background

With the rapid increase of the global ageing population, the age-related disorders, especially Alzheimer’s disease (AD), have emerged as a serious threat to the public health. AD is a neurodegenerative disorder and a leading cause of dementia in the elderly [1]. At present, more than 50 million individuals in the world have AD [2]. The incidence of AD increases with age, especially after age 65. In particular, 1 in 10 people aged ≥ 65 years has AD. Consequently, ageing is considered the primary risk factor for AD. However, the mechanisms underlying the age-related susceptibility to AD remain unclear [3].

The hallmarks of ageing, first proposed in 2013 [4], are defined based on the following three premises: (1) they should be related to age, (2) they can be experimentally manipulated to accelerate ageing and (3) they can stop or reverse ageing upon therapeutic intervention. Referring to López-Otín et al. [5] and Gorgoulis et al. [6], this review summarizes the relationship between AD and ageing based on the following 10 factors: genomic instability, macromolecular damage, epigenetic alterations, deregulated nutrient sensing, mitochondrial dysfunction, cellular senescence, stem cell exhaustion, altered intercellular communication, chronic inflammation and dysbiosis. These factors are closely associated with each other and are critical for understanding the occurrence and development of ageing as well as developing therapeutic strategies for age-related disorders [7].

‘Healthy brain ageing’ refers to the presence of abnormal ageing hallmarks confined to the body’s controllable range without causing harmful effects. In contrast, ‘pathological brain ageing’ refers to the presence of abnormal ageing hallmarks over the body’s controllable range. The progression from early (prodromal) to established AD is caused not only by ageing but also by the pathological deposition of amyloid beta (Aβ) or neurofibrillary tangles (NFTs) in the brain. Studies have demonstrated varying degrees of ageing, such as senescence of different cell types, in both AD patients and animal models [8]. Ameliorating the hallmarks of ageing, such as removing senescent cells, decreases the Aβ- and tau-induced neuropathology and improves memory in AD mice [9–11], suggesting that ageing plays a crucial role in the pathophysiological process of AD. However, the mechanisms underlying ageing and its neuropathophysiological role in AD remain unknown [12].

In this narrative review, we performed literature search in databases PubMed, Web of Science and Embase by using keywords “ageing”, “Alzhermer's disease” and each of the factors such as “genomic instability”. To describe the concept of implementation, Medical Subject Heading (MeSH) terms were used in PubMed and Emtree terms were used in Embase. We summarize the roles of various ageing-related markers in AD to elucidate the impact of ageing on the development of AD and provide novel insights into ageing-related diseases. Moreover, we list novel techniques to study the mechanisms underlying the role of ageing in AD and potential therapies targeting ageing for prevention and treatment of AD.

## Hallmarks of ageing in the course of AD

Ageing is a significant risk factor for neurodegenerative disorders [13]. In healthy individuals, the ageing-associated decline of homeostasis leads to increased cellular stress, including oxidative stress, DNA damage and mitochondrial stress [14]. Senescence may initially occur to maintain homeostasis; however, excessive accumulation of senescent cells results in persistent inflammatory responses and tissue deterioration. The resulting inflammation may cause damage to synapses and lead to cognitive impairment [15, 16]. Patients with AD exhibit tau protein aggregation and Aβ deposition, which lead to ageing of different types of brain cells, resulting in local inflammation [17]. The inflammation, accompanied by accumulation of toxic proteins, contributes to the increased accumulation of stressors within cells, promotes the ageing phenotype and leads to chronic ageing [18]. In this section, we describe the relationship between AD and ageing by summarizing the effects of ageing-related factors on AD.

### Intracellular hallmarks

#### Genomic instability

External stressors such as chemical, physical or biological factors, and endogenous stressors such as DNA replication errors and chromosomal segregation defects, can impair the integrity and stability of the genome [21]. These molecular alterations and the consequent genomic chimerism may contribute to both healthy and pathological ageing [22]. DNA replication errors may result in long-term, irreversible alterations in the final RNA and/or protein output, whereas abnormalities in other biological components are often transient [23].

Unlike proliferating (non-neuronal) cells that experience these changes for a brief period before being replaced, neurons are preserved for life and hence require the ability to withstand long-term damage [24]. Pascarella et al. [25] reported that somatic recombination profiles are altered in Parkinson’s disease and AD. Genomic abnormalities occurring during brain development can greatly increase the risk of AD. Methylazoxymethanol, a major genotoxin found in cycad seeds, induces specific DNA damage and is associated with enhanced tau expression and altered transcription of AD-related genes in primary rat neurons [26]. In AD, genes of DNA repair-associated enzymes are dysregulated in neurons. For instance, histone deacetylase (HDAC) 1, which is involved in DNA repair initiation, is downregulated in neurons in AD [27]. In a recent study, single-cell sequencing of the whole genome revealed increased DNA damage in neurons during neurodegeneration [28]. Neurons are susceptible to DNA damage owing to their non-proliferative nature and high metabolic activity. Excessive DNA damage in neurons is concentrated in genes with differential expression among patients with AD, indicating that dysregulation of gene expression in neurons may be associated with defective DNA repair. Double-strand breaks (DSBs) are the most destructive and severe type of DNA damage that may lead to cell death if not repaired or improperly repaired [29]. Accumulation of DSBs alters the transcription of genes located near the break site, consequently altering neuronal and synaptic function. An imbalance between the accumulation and the repair of DSBs in the brain may lead to neuronal damage as well as learning and memory impairments in AD [30]. Asada et al. [31] found that DSBs and phosphorylated tau frequently co-exist in the cortex of patients with AD. DNA damage may also be involved in the cell state transition under metabolic stress. For instance, DSBs promote the aberrant accumulation of p-tau. Additionally, environmental factors affect the susceptibility of neurons to oxidative stress. Astrocytes, microglia and vascular cells form the surrounding environment of neurons, which release antioxidants or oxidants to block or enhance reactive oxygen species (ROS)-induced damage [32]. Therefore, environmental factors play an essential role in preserving the integrity of DNA and ensuring the survival of neurons.

Almost half of the human genome is composed of repetitive sequences from transposable elements (TEs). Under the action of transposases, TEs can move between genes without relying on the homology of any DNA sequence. Therefore, they are involved in shaping mammalian genomes throughout evolution [33]. Although most TEs have mutated during evolution and have lost the ability to mutate in humans, more than 100 long interspersed elements have retained their full-coding potential and are capable of retrotransposition [34]. Uncontrolled activation of TEs has been observed in various models of neurodegeneration and in brain tissues of patients with neurodegeneration [35]. The CCCTC-binding factor (CTCF), a zinc finger protein that primarily acts as a transcription factor, is involved in gene regulation. The age-associated decrease in CTCF level may affect the expression of Aβ and tau [36]. CTCF is located upstream of many Aβ regulatory proteins, such as PAX6 [37], which regulates GSK-3β and catalyzes Aβ-mediated tau phosphorylation [38]. Defects in CTCF may alter the genome-wide chromatin accessibility and severely impede translation [39]. Therefore, a reduction of CTCF level with ageing may be the first step in the pathophysiological process of AD. Guo et al. [40] sequenced the RNAs of 636 human brain tissues and found that differential expression of numerous retrotransposons was associated with the burden of NFTs and tau-associated active chromatin signatures at various genomic locations. To reduce the transposon-induced replication barriers, cells utilize epigenetic defence mechanisms to limit transposition, including inhibition of heterochromatin formation and production of piwi-interacting RNAs (piRNAs), small RNAs that help to clear transposition factor transcripts [41]. Sun et al. [42] found that the tau deposition-induced absence of piwi and piRNAs and subsequent TE dysregulation seriously hinder the treatment of neurodegenerative diseases. Neurons are postmitotic cells. The neurons will partially acquire the ability to re-enter the cell cycle in AD [43, 44]. Additionally, chromosome 21-specific aneuploidy has been found to be increased in AD brains [45], and functional alterations in cell cycle regulators can influence neuronal migration, axon elongation and pruning, dendrite morphogenesis and synaptic maturation and plasticity, thereby contributing to the development of AD [46].

**Fig. 1:**
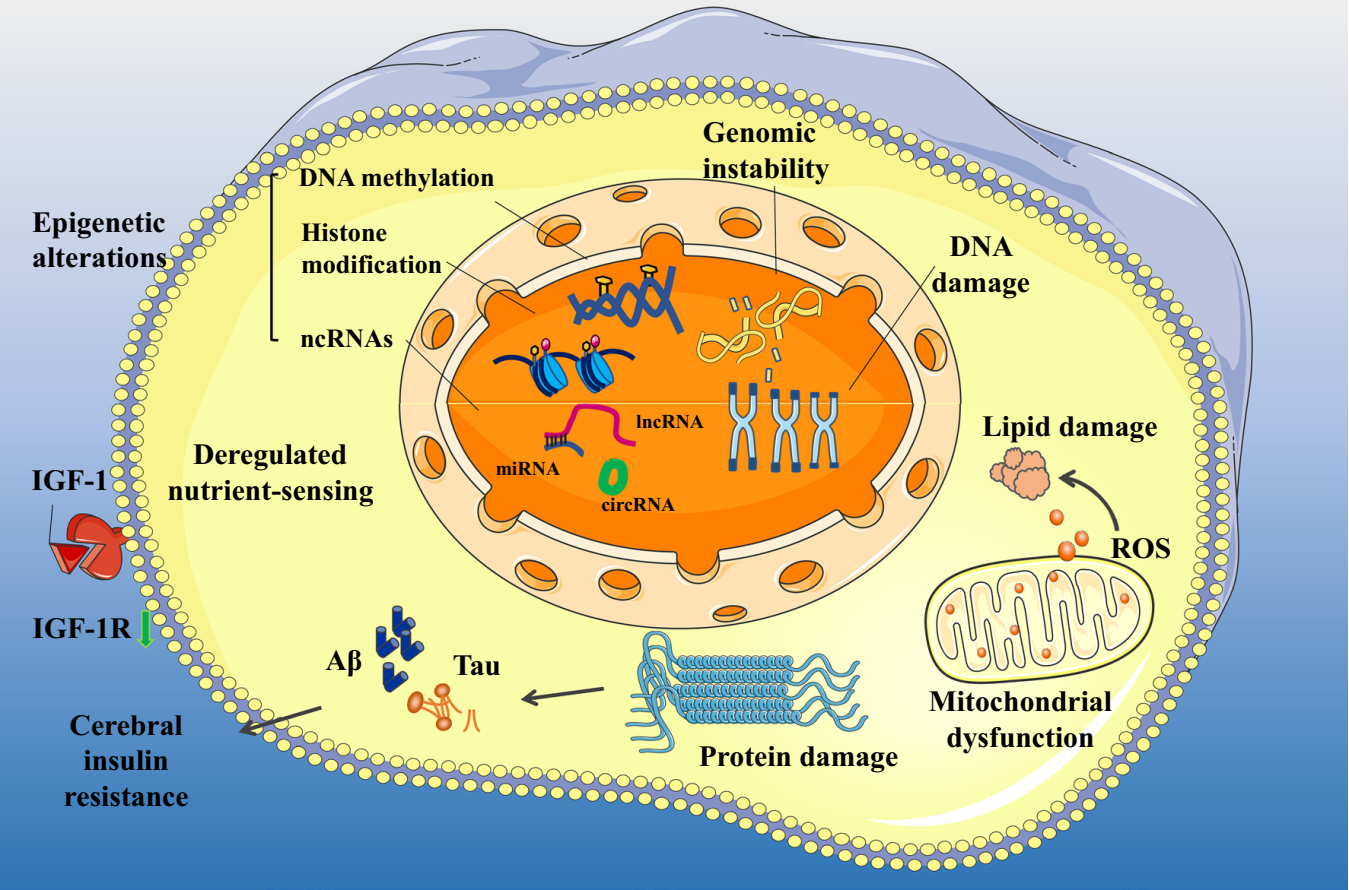
Intracellular hallmarks of ageing in AD. The intracellular hallmarks of ageing include genomic instability, macromolecular damage, epigenetic alterations, deregulated nutrient sensing and mitochondrial dysfunction. Additionally, protein damage in AD mainly manifests as the abnormal aggregation of Aβ and tau proteins, which is related to cerebral insulin resistance and affects nutrient sensing in nerve cells [19]. The green arrow indicates that IGF-1R is downregulated in the brain tissues of patients with AD [20]. *AD* Alzheimer’s disease, *ncRNAs* non-coding RNAs, *lncRNA* long non-coding RNA, *miRNA* microRNA, *circRNA* circular RNA

Altogether, genomic instability remarkably affects the development of AD (Fig. 1) [47]. Understanding the specific mechanisms underlying genomic instability in AD may facilitate the development of preventive strategies for the disease. Although both oxidative DNA damage and deficiencies in repair contribute to genomic instability and are implicated in neurodegenerative processes in AD, the age-related increase in oxidative DNA damage appears to play a predominating role. However, it remains unclear whether the neuronal DNA damage is predominantly induced by autonomous or involuntary processes, and the mechanisms underlying the disturbance of neuronal redox equilibrium that results in cell death and AD-related morbidity, remain unknown. Studies employing single-cell analysis have shown that AD involves complex interactions among all major cell types in the brain. With the development of bioinformatics technology, such as genomics and spatial transcriptomics, the temporal and spatial variations of the entire genome during AD have been successfully mapped. However, the key factors regulating the course of AD remain to be identified in future studies.

#### Macromolecular damage

##### DNA damage

DNA damage at the ends of chromosomes (telomeres) contributes to ageing and age-related disorders [48]. The reverse transcriptase activity of telomerase [49], an active ribonucleoprotein that maintains the sufficient length of telomeres, may prevent telomere loss. However, most somatic cells in mammals do not produce telomerase, resulting in the progressive erosion of telomere sequences from the ends of chromosomes throughout life [50, 51]. Mammals with extremely long telomeres live longer and have improved metabolic health [52]. Pharmacological activation of telomerase has been shown to delay normal ageing in mice.

As individuals continue to age, telomeres get shorter, and approximately 50 nucleotides are lost every cell cycle. Although neurons are post-mitotic cells whose telomeres do not shorten as a result of cell division, they may undergo irreversible DNA damage, leading to senescent cell types and even death [53]. Guan et al. [54] reported that the peripheral leukocytes of AD patients show a decreased proportion of the longest telomeres and a subtelomeric hypermethylation state specific for the shortest telomeres. In another study, Mendelian randomisation (MR) analysis [55] revealed that the genetically predicted longer telomere length is significantly associated with lower levels of Aβ in the cerebrospinal fluid (CSF). Telomerase reverse transcriptase (TERT) proteins, which maintain the length of telomeres, are found in the cytoplasm and mitochondria but not in the nucleus of hippocampal neurons [56]. Iannilli et al. [57] reported that TERT proteins can form complexes with RNA granules and the cell cycle inhibitor p15INK4b in the cytoplasm of hippocampal neurons. During oxidative stress, this complex is disrupted, and different components are released to promote cell senescence. In addition to maintaining telomere length, telomerase also functions to reduce oxidative stress and apoptosis and activate autophagy [58], playing an essential role in brain development and neuronal cells [59]. Both in vitro and in vivo studies have demonstrated that the inhibitory effects of rapamycin on oxidative stress and brain ageing depend on the presence of TERT proteins and its localization to mitochondria. In cells without TERT proteins, the inhibitory effects of rapamycin on the production of ROS are attenuated [60]. Defects in telomerase-rebinding enzymes, such as DNA-PKcs, increase the susceptibility of neurons to AD-associated pathological conditions [61]. Substances that can increase telomerase levels protect hippocampal neurons against Aβ toxicity by increasing the expression of neurotrophic factors and plasticity-related genes, indicating that telomere wear may have an important effect on the aetiology of AD [62]. Li et al. [63] reported that miR-340-5p increases telomere length, delays cell senescence and alleviates the symptoms of AD by reducing POT1 expression in mice.

Altogether, the ageing-induced telomere wear exacerbates DNA damage and contributes to the pathogenesis of AD. TERT proteins can not only increase telomere length, but also exert antioxidant and neuroprotective effects to alleviate the damage caused by telomere loss. Therefore, activating TERT proteins may represent a novel strategy for treating neurodegenerative diseases such as AD.

##### Protein damage

In healthy cells, proteostasis is maintained by a complex network of molecular chaperones, proteolytic processes and related regulatory factors [64]. These factors regulate protein synthesis, polypeptide folding, as well as the maintenance of protein structure and degradation. Multiple external and endogenous stressors that increase with age lead to protein damage. These stressors reduce the activity of the complex network that regulates proteostasis and the integrity of the proteome. Diseases such as AD, Parkinson’s disease and cataracts are caused by the misfolding and accumulation of aggregated proteins, which mostly affect postmitotic cell types such as neurons [65].

Accumulation of misfolded, oxidized, glycosylated or ubiquitinated proteins results in the production of intracellular inclusion bodies and extracellular Aβ, which play a key role in the progression of AD [66]. Errors in protein production become more common with increased age. Pro-adhesive proteins (PCDHs) are the largest subgroup of the calcium-dependent adhesion molecule (cadherin) family. PCDHα, PCDHβ and PCDHγ, three major protocadherin clusters, are abundantly expressed in the brain and play a role in the development of the central nervous system (CNS), cell differentiation and the formation of proper neural circuits [67]. Low expression of protocadherins has been associated with dysregulation of dendritic shape and branching. PCDHα is essential for learning and memory [68]. Consistently, Li et al. [69] reported that PCDHγC5 is upregulated in Aβ-treated hyperexcited neurons and transgenic APP/PS1 mice.

As a component of the network that regulates protein turnover, autophagy is closely related to the management of cell quality [70]. The inflammatory response and oxidative stress induced by Aβ may reduce macroautophagy. In addition, the decrease of autolysosome-driven acidification of neurons precedes the extracellular deposition of Aβ. Microglial autophagy maintains the α-synaptic nucleoprotein homeostasis, thereby regulating neuroinflammation and delaying the progression of AD [71]. Atg7 is a crucial regulator of autophagosome biogenesis and regulates lipid metabolism and neuroinflammation in microglia [72]. Deletion of the microglia-specific Atg7 results in the transformation of microglia to a pro-inflammatory phenotype in vivo and the activation of inflammasomes in vitro. Xu et al. [73] demonstrated that deficiency of Atg7 in microglia exacerbates tau pathology and its spreading in neurons. Chaperone-mediated autophagy (CMA) is a selective mode of autophagy that degrades neurodegeneration-related proteins [74]. Inhibition of CMA disrupts protein homeostasis and leads to changes in neuronal function. Suppression of CMA in a mouse model of AD has been shown to exert synergistic effects on proteins at risk of aggregation, thereby increasing the susceptibility to neurological disorders and accelerating disease progression. However, chemically enhancing CMA ameliorates characteristic pathology in two mouse models of AD [75]. Therefore, CMA plays an essential role in regulating protein homeostasis in neurons.

Recent studies analyzing whole-proteome changes during ageing have shown that protein homeostasis is involved in the entire pathological process of AD. In addition to classical Aβ and tau deposition, proteins such as cadherins greatly affect the development of AD. Changes in protein homeostasis are not limited to an increase or decrease in protein level. Conformational heterogeneity may considerably alter the functions of proteins such as chaperones. Pharmaceutical agents that improve protein homeostasis can be used to delay the onset of age-related diseases associated with proteome degradation [76].

##### Lipid damage

Lipids play an essential role in maintaining cell membrane integrity, energy production and signal transduction. Altered lipid metabolism has been observed in some ageing-related diseases. Impairment of lipid metabolism can induce AD by promoting Aβ deposition. APOE, a cholesterol/lipid transporter in the CNS, is the most influential risk factor for AD [77, 78]. The ε4 allele of the *APOE* gene (compared with the most common ε3 allele) is the strongest genetic risk factor and the relatively rare *APOE* ε2 allele is the strongest protective genetic factor against sporadic AD [78]. APOE and ABCA1 regulate cholesterol transfer into and out of brain cells, respectively. APOE/ABCA1 imbalance causes lipid accumulation in the brain [79]. Accordingly, deletion of the *ABCA1* gene reduces APOE lipidation and increases Aβ deposition in the brain [80]. A rare mutation (R47H) in triggering receptor expressed on myeloid cells 2 (TREM2), a microglial surface receptor, has been associated with a substantially high risk of AD. TREM2 can activate microglia to phagocytose Aβ by sensing various anionic and zwitterionic lipids known to be associated with fibrous Aβ in lipid membranes [81]. Cholesterol metabolism in the brain is often impaired during ageing and AD [82]. Dysregulation of lipid metabolism is more pronounced in older individuals than in younger individuals. In a previous study, older mice were found to have a more persistent inflammatory response than younger mice after myelin injury. This phenomenon was attributed to the accumulation of excess myelin fragments in aged phagocytes, which resulted in the formation of cholesterol crystals that stimulate inflammasomes [83]. Wiley et al. demonstrated that senescent cells activate the biosynthesis of several oxidized lipids that promote the senescence-associated secretory phenotype (SASP) [84]. Quantitative analysis of these oxidized lipids may be an approach to assessing ageing. Several studies have shown that cerebral lipid peroxidation increases regionally in patients with AD. Notably, lipid metabolism disorder is frequently accompanied by neuroinflammation, and lipid damage is closely related to mitochondrial dysfunction during ageing [85]. The brain relies heavily on mitochondrial oxidative phosphorylation (OxPhos) to degrade fatty acids and maintain lipid homeostasis in astrocytes [86]. Abnormal OxPhos may induce the accumulation of lipid droplets in astrocytes. Moreover, when the accumulation of fatty acids exceeds the OxPhos capacity of astrocytes, the upregulated acetyl-CoA would induce astrocyte activation by enhancing the acetylation and activation of STAT3. Eventually, reactive astrocytes enriched with lipids induce neuroinflammation and oxidative stress [87]. Studies investigating the specific targets of lipid damage are limited. An in-depth understanding of the relationship between lipid damage and AD may help elucidate the pathogenesis of AD and develop more effective treatment methods.

#### Epigenetic alterations

Epigenetic changes that lead to ageing include altered DNA methylation patterns, abnormal histone post-translational modifications, abnormal chromatin remodelling and dysfunction of non-coding RNAs (ncRNAs) [88]. These regulatory, and often reversible, alterations affect gene expression and other cellular processes, leading to the development and progression of age-related diseases such as cancer, dementia, metabolic syndrome and bone disease [89].

##### DNA methylation

DNA methylation occurs when a hydrogen atom in a DNA strand is replaced by a methyl group. Methylation is catalyzed by DNA methyltransferases and typically occurs on the fifth carbon atom of the cytosine residue (5mC) of CpG and non-CpG (CpA, CpC and CpT) dinucleotides [90]. CpG dinucleotides are predominantly methylated in all organs of vertebrates, with approximately 80% of CpG sites possessing 5mC. The number of methylated CpH (mCpH) sites in the brain increases with age. Enhancers with hypermethylated CpH sites are associated with genes functionally enriched in immune responses, some of which are related to neuroinflammation and degeneration. A previous Whole Epigenome Association Study found that early-stage and severe AD patients have more genes with altered DNA methylation levels in the hippocampus, entorhinal cortex, dorsolateral prefrontal cortex (PFC) and cerebellum than control patients [91]. These genes have been associated with AD susceptibility and are involved in different pathological processes in neuronal or non-neuronal cells, such as Aβ pathology (*APP*, *ABCA7*), tau protein pathology, inflammation (*IL-1β* and *IL-6*) and abnormal protein metabolism (*RPL13* and *HOXA3*) [92]. Han et al. [93] reported that the expression of the m6A methyltransferase METTL3 was increased and that of the m6A demethylase FTO was decreased in mice with AD. These findings suggest that m6A modification is enhanced during the development of AD.

Altogether, DNA methylation is implicated in ageing and AD (Fig. 1). AD-specific de novo methylation of numerous previously unmethylated sites plays a vital role in the development of AD [94]. Although the precise mechanisms underlying aberrant DNA methylation remain unclear, oxidative DNA damage has been reported to play a significant role. Given the close relationship between DNA methylation and damage responses, we hypothesize that AD-specific methylation patterns result from and reflect more oxidative damage, which necessitates substantial repair and eventually results in more errors. DNA methylation involves the coordination of different epigenetic markers, such as H3K18ac and H3K23ac (involved in the regulation of histone acetylation), which collectively affect gene transcription. Although the relationship between DNA methylation and histone modification remains elusive, the two mechanisms have been reported to involve shared regulatory pathways.

##### Histone modification

Histone modification is another essential epigenetic mechanism in which histones are covalently altered to modify the intrinsic chromatin structure and influence gene expression. In gene promoters and enhancers, methylation and acetylation of positively charged lysine (K) and arginine (R) residues are the most common histone modifications [95]. Histone modifications are dynamic and reversible and are regulated by enzymes known as writers (such as histone acetyltransferases or methyltransferases) and erasers (such as histone deacetylases or demethylases). An imbalance between writers and erasers results in the dysregulation of transcription and the alterations of epigenetic markers in many brain diseases. Dysregulation of histone methylation and acetylation has been associated with the pathogenesis of early-stage AD. HDACs are well-known enzymes that remove acetyl groups from lysine residues in the amino-terminal domain of histones [27]. Dysfunction of HDACs (HDAC1, HDAC2, HDAC3 and HDAC6) has been associated with cognitive impairment. The expression of HDAC1 and HDAC2 is decreased in the PFC and hippocampus in advanced AD [96]. The epigenome-wide H3K9 acetylation (H3K9ac) and H3K27ac levels are increased in AD [97]. In addition, upregulation of H3K9ac in the PFC and temporal lobe and its downregulation in the hippocampus may play a major role in the pathogenesis of AD, indicating that H3K9ac is a suitable therapeutic target for AD [98]. Recent studies have demonstrated that HDAC inhibitors improve memory and synaptic function in cellular and animal models of AD [99]. Alterations of many HDACs can affect ageing indicators in AD. As class III HDACs, sirtuins play an important role in regulating various physiological processes, including apoptosis, DNA repair, inflammatory responses and metabolism, and their function depends on nicotinamide adenine dinucleotides (NAD+) [100]. Zhao et al. [101] reported that the SIRT1–FOXO1/3–PINK1–Parkin pathway regulates mitochondrial autophagy to reverse mitochondrial dysfunction and exerts anti-AD effects.

Altogether, histone acetylation indicators, namely, H3K9ac and H3K27ac, have altered levels in AD, which may result in improper or excessive activation of implicated genes, leading to cell death. However, the levels of H3K9ac and H3K27ac in other regions of the brain in AD and the mechanisms underlying their aberrant levels remain unclear. Although histone H3 lysine 4 trimethylation (H3K4me3) and H3K9me2 (histone H3 lysine 9 dimethylation) are enriched in genes associated with cellular defense and memory development, in-depth information regarding the relationship between these modifications and genes is lacking [102]. Understanding the precise mechanisms underlying histone modification in response to DNA damage and changes in the levels of related markers at different disease stages may facilitate the development of methods for controlling histone modification in the treatment of AD.

##### ncRNAs

ncRNAs are a family of non-protein-coding transcripts that are subdivided into small ncRNAs (< 200 nucleotides) including microRNAs (miRNAs; approximately 22 nucleotides); long ncRNAs (lncRNAs; > 200 nucleotides); and circular RNAs (circRNAs) [103]. Approximately 80% of the human genome is transcribed as non-coding transcripts, whereas only 2% codes for proteins. ncRNAs are particularly prevalent in the CNS. Approximately 70% of miRNAs and 40% of lncRNAs are expressed in the brain. These non-coding transcripts are mostly found in the nucleus, indicating epigenetic control as their principal function [104]. Most ncRNAs are expressed with precise temporal and spatial patterns in the CNS. Dysregulation of complex multitasking ncRNAs (particularly miRNAs and lncRNAs) is related to the fundamental pathogenic phenotype of AD. The lncRNAs BACE1-AS, BC200 and NEAT1 have been associated with the regulation of Aβ aggregation, whereas the lncRNAs repressor element 1-silencing transcription factor (REST), RPPH1 (Ribonuclease P RNA Component H1), MALAH1, MEG3, miR-125-b, miR-361-3 and miR-122 have been associated with cellular apoptosis [105]. REST is an epigenetic master regulatory gene that serves as a marker of ageing in human cortical and hippocampal neurons, and its level is strongly associated with AD and life expectancy. REST binds to hundreds of genomic sites and controls numerous neuron-specific coding and non-coding genes. It forms complexes with histone-modifying enzymes and other proteins involved in chromatin remodelling [106]. These complexes may form a big complex with HOX antisense intergenic RNA (HOTAIR) and polycomb repressive complexes 2 (PRC2) [107]. PRC2 is present in almost all eukaryotic cells and is responsible for the epigenetic control of > 2000 genes on 10 chromosomes. Additionally, it is responsible for the alterations of 5%–10% of histones to the H3K27me3 form. REST increases the local H3K27me3 levels through PRC2. Given that H3K27me3 is important for DNA damage response and is associated with AD, HOTAIR may play a role in DNA damage response and AD pathogenesis by interacting with REST and PRC2 to regulate the levels of epigenetic markers, including H3K27ac, H3K27me3 and H3K4me3.

ncRNAs are involved in the formation of Aβ plaques, tau phosphorylation and inflammation, three major pathophysiological processes in AD (Fig. 1) [108]. However, the interactions among various ncRNAs remains unclear. Relatively comprehensive lncRNA-related competing endogenous RNA networks have been constructed based on brain tissue samples from APP/PS1 mice [109]. In future studies, these networks can be combined with multiple ncRNAs to gain novel insights into the diagnosis and treatment of AD.

#### Mitochondrial dysfunction

In addition to serving as the powerhouse of cells, mitochondria can potentially trigger inflammatory responses [110]. Mitochondrial function deteriorates with ageing due to various inter-related mechanisms, including accumulation of mitochondrial DNA mutation, dysregulation of the respiratory chain complex owing to the loss of protein homeostasis, reduced organelle turnover and alterations of mitochondrial dynamics. These alterations decrease the productive efficiency of mitochondria in cells, increase the production of ROS and may lead to abrupt mitochondrial membrane perforation, resulting in inflammation and cell death [111].

Although mitochondrial dysfunction is involved in each step of disease progression, the dysfunction can be observed before the occurrence of pathophysiological changes and targeted for early therapeutic intervention [112]. Localized hypometabolism in patients with AD can reveal initial signs of mitochondrial failure [113]. Changes in glucose metabolism in the brain can reflect alterations in energy metabolism in patients with AD [114]. Mitochondrial function can be affected by the interactions between AD-related neuropathological markers (APP, Aβ and tau) and genetic risk factors (APOE, etc.) [115–117]. A previous study showed that a low intracellular NAD^+^/NADH ratio is involved in the mitochondrial dysfunction-related senescence, and prevents the IL-1-mediated SASP through AMPK-mediated activation of p53 [118]. Swerdlow et al. have proposed the mitochondrial cascade hypothesis in AD [119]. In particular, addition of Aβ to mDNA-free neuronal cells does not induce cell death, indicating that the mitochondrial cascade reaction is important for triggering Aβ toxicity [120]. Mitochondria respond to changes in energy through continuous fission and fusion during AD [121]. Mitochondrial complex I has been shown to directly affect pTau deposition and energy homeostasis in mice [122]. As an important part of the mitochondrial cascade hypothesis, mitochondrial calcium overload is prevalent in AD, and the absence of the mitochondrial sodium/calcium exchanger can directly induce AD-like pathological characteristics [123]. Mitochondrial dysfunction may be secondary to abnormal deposition of neuropathological markers (APP, Aβ and tau), or a primary mitochondrial cascade may directly disturb vital brain functions [119].

The energy produced by mitochondria is required by astrocytes to perform neuroprotective functions. Interfering with mitochondrial homeostasis in astrocytes and the impaired ability of mitochondria to produce ATP can negatively affect neural function. In ageing or other pathological conditions, alterations in mitochondrial function in astrocytes may precede or accompany neuronal injury [124]. Mitochondria are the primary source of ROS, and the ROS generated by dysfunctional mitochondria may cause damage to DNA, lipids and proteins [125]. Park et al. reported that an increase in NADPH oxidase-4 level aggravates oxidative stress in human astrocytes through the production of mitochondrial ROS, fragmentation of mitochondria and suppression of cellular antioxidant mechanisms, thereby enhancing the ferroptosis of astrocytes [126].

The mitochondrial unfolded protein response (UPRmt) maintains protein homeostasis in mitochondria by initiating the transcriptional activation of mitochondrial chaperone proteins and proteases [127]. Inhibition of UPRmt is involved in several human disorders, including age-related diseases such as AD, Huntington’s disease and Parkinson’s disease [128]. Stimulation of UPRmt and high expression of UPRmt-related components have been associated with an extended lifespan. Shen et al. [129] reported that inhibition of UPRmt enhanced the toxic effects of Aβ_25-35_ on SHSY5Y cells. Sirtuin (SIRT) 3 is a deacetylase that is localized in mitochondria and regulates acetylation levels in mitochondria. SIRT3 expression and activity are significantly decreased in the hippocampal mitochondria in APP/PS1 mice [130]. The decreased expression is closely related to mitochondrial dysfunction and UPRmt [131]. Hou et al. [132] demonstrated that Honokiol, an anti-AD component derived from the bark of *Magnolia officinalis*, ameliorates the Aβ-induced mitochondrial dysfunction in a SIRT3-dependent manner. In addition, Sorrentino et al. [133] found that enhancement of mitochondrial proteostasis significantly delays the Aβ-associated symptoms in AD.

Given that mitochondria are important sites of cellular energy production, mitochondrial failure is common in neurodegenerative diseases, especially AD [134] (Fig. 1). Therefore, mitochondrial function plays an essential role in maintaining health, and progressive degradation of mitochondria can lead to ageing phenotypes. Treatment strategies targeting different elements of mitochondrial dysfunction involve the inhibition of mitochondrial processes associated with oxidative stress and apoptosis [135]. Early oxidative stress has been observed in the brains of AD patients and animal models. Therefore, antioxidant treatment may be beneficial [136]. A study demonstrated that the mitochondria-targeted antioxidant catalase (MCAT) transgenic AD mice (AD-MCAT) have longer survival and lower expression of neuropathological markers of AD (APP, oligo-Aβ) than AD mice [137]. These findings suggest that directing antioxidants to mitochondria is a viable strategy for effective treatment of ageing and AD. MitoQ, an antioxidant that targets mitochondria, reduces Aβ-induced neuronal cell death and oxidative stress in the cortical neurons of mice and alleviates AD-like symptoms [138, 139]. Altogether, mitochondrial oxidative stress plays an important role in the development of several pathological characteristics of AD, and an early intervention may be the key to successful therapies [140].

#### Deregulated nutrient sensing

Nutrient-sensing networks are highly evolutionarily conserved. Extracellular ligands, such as insulin and insulin-like growth factors, interact with receptor tyrosine kinases and intracellular signal cascades [141] to regulate several cellular processes, including autophagy, mRNA and ribosome biogenesis, protein synthesis, glucose and lipid metabolism and mitochondrial biogenesis [142]. Regulating the expression of genes involved in nutrient-sensing networks has been shown to extend survival in different animal models.

Insulin resistance is common in patients with early-onset AD. It impairs vascular function by affecting the clearance of Aβ peptides, phosphorylation of tau proteins, vascular reactivity, lipid metabolism and inflammation. Therefore, restoring insulin function in the brain may benefit patients with AD [143, 144].

Glucagon-like peptide 1 (GLP-1) is an incretin hormone released from enteroendocrine L-cells upon nutrient ingestion and also released by *proglucagon*-expressing neurons in the nucleus of the solitary tract (NTS) of the hindbrain. A recent study showed that GLP-1 limits glucose uptake and enhances beta-oxidation in cultured astrocytes, while deletion of GLP-1 receptor results in an adaptive stress response that improves systemic glucose homeostasis and memory development [145].

The Ras–Raf–Mek–Erk pathway is another important nutrition-related pathway involved in the development of AD. Activation of this pathway leads to the activation of transcription factors that stimulate the transcription of many cell cycle regulators and are associated with apoptosis. Suppression of the Ras/Mek/Erk signalling pathway may alleviate the AD-related cognitive impairment. Mai et al. [146] reported that deletion of *DHCR24* resulted in hyperphosphorylation of tau in astrocytes through activation of the lipid raft-dependent Ras/Mek/Erk signalling pathway, which is involved in the aetiology of AD. Oxygen homeostasis is essential for maintaining cell function. 2-Oxoglutarate and ferrous iron-dependent oxygenases (2OGDDs) are hypoxia sensors that play an essential role in regulating oxygen homeostasis [147]. Several 2OGDD-related cellular processes are altered in the brains of AD patients. Therefore, 2OGDDs may act as key regulators of the transition from normal brain ageing to AD.

Altogether, deregulated nutrient sensing, with altered insulin signalling as its core, is a crucial component involved in the pathogenesis of AD (Fig. 1). The hippocampus, which plays a crucial role in memory development and consolidation, has wide distribution of insulin receptors and is a target of insulin resistance. Insulin signalling pathways promote neuronal survival and synaptic plasticity and contribute to advanced brain functions, such as cognition [148]. Disturbance of insulin signalling pathway increases the risk of AD. Given that insulin sensitivity is strongly influenced by environmental factors, the integrated role of biological, social and lifestyle factors in the progression of AD highlights the potential of psychological and behavioural interventions to prevent or delay AD [143].

### Cellular hallmarks

#### Cellular senescence

**Fig. 2:**
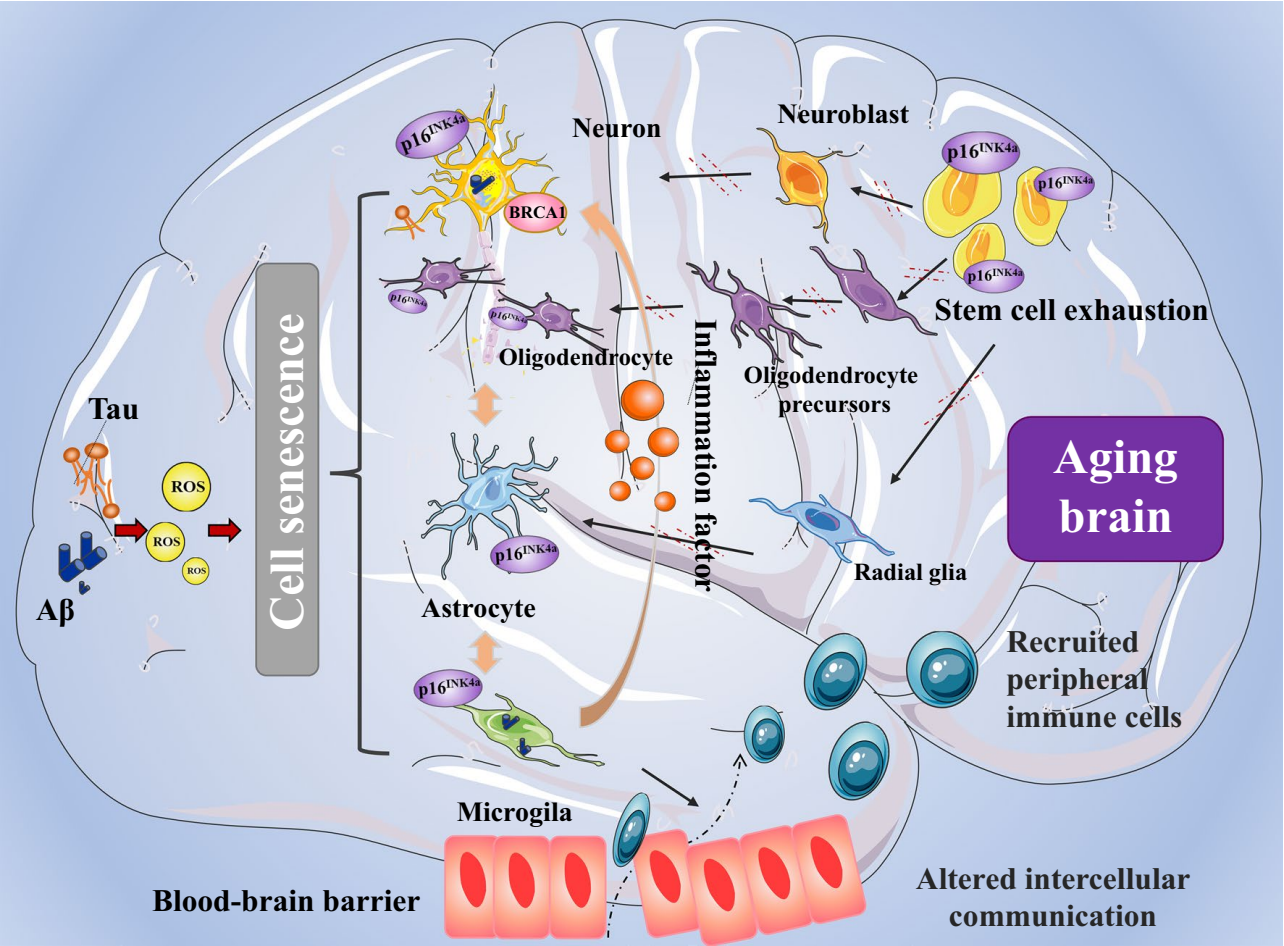
Cellular hallmarks of ageing in AD, including cellular senescence, disabled macroautophagy, stem cell exhaustion and altered intercellular communication. ROS activated by Aβ and tau can cause stress-induced cellular senescence [10, 152]. The senescent cells exhibit significant dysfunction and abnormal intercellular communication. Ageing microglia release more pro-inflammatory factors and have impaired phagocytic function [153]. Simultaneously, stem cell exhaustion inhibits the transformation of stem cells into neurons, astrocytes and oligodendrocytes [154]. *AD* Alzheimer’s disease, *ROS* reactive oxygen species, *P16*^*INK4a*^ a marker of cellular senescence

Cellular senescence, which includes replicative senescence and stress-induced premature senescence, is the permanent cell cycle arrest [149]. The primary hallmarks of senescent cells include loss of the proliferative or regenerative capability, altered metabolic function, resistance to apoptosis and secretion of various disease-causing active molecules known as SASP factors [150]. In most cases, SASP is considered harmful and would lead to abnormalities in body function and age-related diseases. Studies in humans and animals have indicated a crucial role of cellular senescence in the onset of several age-related diseases [151]. Pre-clinical and clinical studies have indicated that cellular senescence plays a crucial role in the onset of several age-related diseases, including AD [10] (Fig. 2).

##### Neuronal senescence

Age is the basic biological factor affecting subcortical and hippocampal symmetry [155]. As brain function constantly changes with age, alleviating the pathological damage caused by brain ageing is a promising strategy for improving cognitive function [156]. Mertens et al. reported that age-dependent cellular changes impair the functions of neurons in AD [157]. In another study, single-cell transcriptomic analysis revealed that neurons derived from patients with AD exhibited ageing-like features, including metabolic dysfunction and elevated levels of pro-inflammatory markers [158]. In AD, senescence of neurons may initially result from abnormal stimulation of proteins, such as Aβ and tau oligomers [159]. Wei et al. [160] reported that the level of the neuronal ageing-related marker p16 was significantly higher in 5 × FAD mice, indicating an evident ageing phenotype. In vitro experiments demonstrated that Aβ_1-42_ induced the production of p16 and aggravated cell ageing. Accumulation of tau-containing NFTs has been associated with brain ageing and cognitive decline. Musi et al. [10] and Dehkordi et al. [161] performed transcriptomic analysis on NFT-containing neurons from autopsied brain tissues of patients with AD and found that the expression profiles were consistent with those observed in cellular senescence. Metabolic abnormalities, changes in cholinergic receptors and neuroinflammation, which are risk factors for cell senescence, may occur in the brains at early stage of AD [162]. Senescent neurons may further suffer increased deposition of Aβ, creating a vicious feedback loop. Interleukin-like EMT inducer (ILEI, also known as the family with sequence similarity 3C) binds to the γ-secretase complex to suppress Aβ production without blocking γ-secretase activity. ILEI is widely expressed in normal neurons and ependymal cells, and its expression level peaks after birth and decreases with age. In a previous study, the number of ILEI-immunoreactive neurons was found to be lower in patients with AD than in age-matched control individuals [163]. The decreased expression of ILEI in ageing neurons may further induce the accumulation of Aβ in the brain and aggravate AD.

Brain ageing in AD appears to behave differently from normal ageing. Neurons lose the ability to reproduce during normal ageing, whereas numerous neurons re-enter the cell cycle during pathological ageing in AD, indicating the recovery of their reproductive potential [164]. However, these neurons often lose their normal function, coexist with cells characterised by ageing and are co-involved in the dysfunction of the ageing brain. Raina et al. [165] reported increased expression of MORF4 (a transcription-activating protein) in many neurons found in vulnerable brain regions in AD, indicating the re-entry of these neurons into the cell cycle. BRCA1, a known cell cycle regulator, is strongly expressed near NFTs in the brains of patients with AD. However, its expression is significantly reduced in normal-ageing brain tissues in clinical settings [166]. This difference in BRCA1 expression may explain the absence of neuronal re-entry into the cell cycle in the normal ageing brain and the abundance of neurons entering the cell cycle in AD. Therefore, these neurons may be studied to reveal the differences between normal ageing and pathological ageing in AD. However, to date, no studies have identified the mechanisms underlying the re-entry of neurons into the cell cycle in AD.

##### Astrocyte senescence

The burden of p16^INK4a^ (a senescence biomarker)-positive astrocytes in the frontal cortex is substantially greater in patients with AD than in healthy individuals of the same age [167]. On the one hand, abnormal protein deposition, such as tau accumulation, induces the ageing of astrocytes; on the other hand, ageing astrocytes produce neurotoxic factors to induce inflammation and oxidative stress, further aggravating the development of AD [168]. The age-dependent increase in the expression of GFAP and astrocyte hypertrophy may reflect the potential toxicity of astrocytes [169]. Limbab et al. [170] reported that the X-irradiation-induced senescence of human astrocytes led to down-regulation of glutamate and potassium transporter genes in vitro. This downregulation caused neuronal cell death when the astrocytes were co-cultured with normal neurons. Aged astrocytes develop a neuroinflammatory A1-like reactive phenotype, and aged brains have more A1 reactive astrocytes in response to neuroinflammatory inducers, such as lipopolysaccharides. When the A1-like reactive astrocytes lose their normal function, they produce complement components and release a toxic factor that kills neurons and oligodendrocytes, leading to cognitive decline in vulnerable regions of the brain during normal ageing [171]. Turnquist et al. demonstrated a high abundance of senescent astrocytes in the brain tissues of patients with AD. These astrocytes exhibit the same SASP as senescent astrocytes in vitro [172]. Furthermore, Bussian et al. reported that the MAPT^P301S^PS19 mice with tau-dependent neurodegenerative disease have a high abundance of P16^INK4A^-positive senescent astrocytes, and clearance of these astrocytes significantly reduces the formation of NFTs [173].

Therefore, astrocyte senescence may play an important role in the progression of AD, and effective prevention of astrocyte senescence may represent an important therapeutic strategy for AD.

##### Microglial senescence

The continuous proliferation of microglia is a hallmark of AD. Microglial proliferation is significantly different between healthy and ageing brains [174]. A recent study showed that microglia undergo replicative senescence in the APP/PS1 model, which is dependent on early and sustained proliferation of microglia and characterized by increased senescence-associated β-galactosidase activity, age-related transcription signature and telomere shortening [153]. Microglia in patients with AD also exhibit an ageing phenotype [175]. As SASP factors are composed of various pro-inflammatory factors including cytokines, growth factors, angiogenic factors and soluble proteins, as well as extracellular vesicles [176], it is difficult to distinguish between the inflammatory activation state and the ageing state of inflammatory cell types such as microglia. The phagocytic function of microglia is usually enhanced at an inflammatory activation state [177] while substantially reduced at the ageing state. Hu et al. [178] reported that Aβ_1-40_ induced microglial senescence in vitro. Increased expression of CD38 in ageing microglia is associated with energy metabolism disorder and neuroinflammation. Moreover, Aβ substantially reduces the mRNA expression and protein level of SIRT1 (a major regulator of ageing) as well as the mRNA expression and translocation of NRF2, a critical transcription factor in inflammatory responses, resulting in impairment of the phagocytic function of microglia [179]. Notably, microglia tend to become proinflammatory and chemotactic during ageing. The aged microglia promote T-cell infiltration and exacerbate neuroinflammation in the brain by releasing chemokines [180]. In addition, the persistent inflammatory activation state can induce ageing, resulting in decreased phagocytic function of microglia [181]. In some cases, inflammation co-exists with the aging state. Additionally, aged microglia have abnormal metabolic activities [182]. The receptor TREM2 on microglia acts as a sensor for various lipids, whereas defects in TREM2 affect the Akt signalling pathway for lipid metabolism [183]. Microglia require prompt remodelling of the plasma membrane to monitor local changes in brain function and control neuronal activity. Yoo et al. found that replacing mutant microglia with circulation-derived myeloid cells following haematopoietic cell transplantation restores the function of Trem2 and improves cognitive function in a Trem2 mutant mouse model of AD [184]. This finding indicates that microglia may be targeted for alleviating the symptoms of AD.

##### Senescence of other cells

Decreased white matter volume and function is an important sign of brain ageing. Senescence of oligodendrocytes, a critical component of white matter, has been associated with decreased oligodendrocyte status and total oligodendrocyte density. By single-cell RNA sequencing, Kaya et al. [185] observed a lymphocyte-dependent, IFN-responsive microglial subpopulation localized near CD8^+^ T cells in the ageing white matter. CD8^+^ T cells can inhibit glial cell loss; however, the underlying mechanisms remain elusive. Peripheral immune cells play a crucial role in age-related neurodegenerative diseases [186]. Enhanced lymphocyte migration is involved in the amplified response of the ageing brain to acute exogenous inflammatory stimuli. However, the molecular regulation of blood–brain barrier (BBB) and the interaction between peripheral immune cells and the CNS during ageing warrant further investigations [187]. The age-related phenotypic changes of cerebrovascular endothelial cells lead to abnormal cerebral blood flow and disruption of the BBB, contributing to vascular cognitive impairment. Kiss et al. [188] used single-cell sequencing and found that the number of ageing endothelial cells increases by ~ 10% with advanced ageing. Bryant et al. reported that the expression of the SASP-related genes *SERPINE1* and *CXCL8* in microvascular endothelial cells of the frontal cortex was 2 times higher in patients with AD than in healthy individuals of the same age [189].

In summary, AD is closely associated with cellular senescence [164]. Exposure to toxic forms of Aβ or aggregated tau proteins can lead to senescence of several types of brain cells. Selective elimination of senescent cells can inhibit Aβ deposition and NFT formation in the brains of mice, thereby improving memory and learning [164, 190]. However, different types of cells have unique forms of ageing and may play different roles in ageing-related diseases. In future studies, single-cell RNA sequencing may be used to examine the effects of ageing on different types of brain cells and the efficacy of various anti-ageing therapies in distinct tissues.

#### Stem cell exhaustion

Adult stem cells are crucial for tissue homeostasis and regeneration. Stem cell exhaustion defined as the quantitative and qualitative reduction in stem cell activity during life, is considered one of the causes of ageing [191]. Unlike the adaptability of resident stem cells under normal homeostasis, the plasticity during injury diminishes with ageing [192].

Neural stem cells (NSCs) play an important role in physiological homeostasis and pathological repair of the CNS [193]. Because stem cells are highly delicate, their homeostasis may be disrupted under several circumstances, directly leading to decreased proliferation, which is one of the most prominent features of senescent stem cells. NSCs are usually deficient in differentiation and DNA-repair abilities in neurodegenerative disorders. Increased levels of p16^INK4a^, a marker of cell senescence and cell cycle arrest, and accumulation of damaged DNA, are closely associated with a decrease of stem cells during ageing [194]. The normal use of human stem cells (such as NSCs) requires maintenance of their nuclear structure and chromatin homeostasis. Nuclear reprogramming to pluripotency can revert both the age and the identity of any cell to that of an embryonic cell [195]. The H3K4me3 domain has been demonstrated to enhance the self-renewal and differentiation capabilities of mouse NSCs [196]. Although epigenetic dysregulation is associated with changes in stem cell populations throughout life, the mechanisms through which particular epigenetic markers and their regulators participate in stem cell senescence remain unknown and warrant further investigation. Protein homeostasis plays a crucial role in maintaining stem cell function. The transmembrane protein Ttyh1 is necessary for the survival of NSCs [197]. Based on an integrative bioinformatic analysis, Cao et al. [198] found that Ttyh1 regulates the resting and activation states of NSCs through Ca^2+^/NFATc3. Single-cell RNA sequencing of young and aged neurogenic niches revealed a decline in the number of activated NSCs in the ageing brain [199]. Lee et al. [200] reported that Aβ oligomers regulate the proliferation, differentiation and migration of human NSCs through glycogen synthase kinase-3β. The SASPs of other cells can induce the senescence of stem and progenitor cells, leading to poor tissue maintenance and reduced regenerative potential of tissues, eventually resulting in inflammation [201].

Oligodendrocyte progenitor cells (OPCs) are an important class of brain stem cells. In the brains of patients with AD, Aβ-associated OPCs expressing Olig2 and ng2 show increased expression of p21/CDKN1A and p16/INK4/CDKN2A and increased age-related β-galactosidase activity, exhibiting an ageing-like phenotype [154]. Direct exposure to Aβ induces senescence of OPCs in vitro. In mice with AD, senolytic treatment selectively removes senescent OPCs from the plaque environment, which reduces neuroinflammation and Aβ load and improves cognition. As an early event of AD, myelin fragmentation is closely related to the normal function of OPCs. Myelin-coated axons are maintained by a dynamic demyelinating–remyelinating equilibrium, which involves the coordinated action of OPCs and microglia [202]. OPCs develop into oligodendrocytes, which produce myelin. Microglia eliminate myelin debris and produce signalling molecules that stimulate the generation of oligodendrocytes from OPCs. However, as OPCs age, their differentiation rate decreases, diminishing their regenerative capacity. A previous study showed that the staining intensity of myelin basic protein and the density of OPCs in the hippocampus decrease with age in 3 × Tg AD mice. In addition, OPCs were found to undergo complicated age-related remodelling [203]. Degradation of OPCs is an early pathological indication of AD and represents a potential mechanism that drives myelin loss and cognitive impairment (Fig. 2).

Adult stem cells are essential for maintaining optimal tissue function. Many factors, including genomic instability, telomere wear, epigenetic alterations, cellular ageing, mitochondrial malfunction, protein homeostasis and altered intercellular communication, contribute to the age-related reduction in stem cell function. Therefore, stem cell regeneration may reverse the ageing phenotype in vivo, and understanding the underlying mechanisms may help to identify novel therapeutic targets for ameliorating age-related phenotypes and prolonging survival in humans. Some recent studies have shown that metabolites and their associated epigenetic regulators may reverse the ageing phenotype of adult stem cells. Regenerative stem cell ageing directly affects epigenetics and gene expression. Resveratrol protects adult stem cells from premature ageing and considerably extends the survival of mice by enhancing the interaction between SIRT1 and p53 in mice [204]. Exogenous stem cell therapies aim to restore cognitive function by introducing stem cells to repair the degraded neural networks. Based on the paracrine ‘bystander’ mechanism, these stem cells can be used as delivery systems through production or induced production of neuroprotective growth factors. Alternatively, functional recovery can be achieved by inducing the differentiation of stem cells to compensate for the degenerative neuronal circuits. Exogenous stem cell therapy has been shown to alleviate the cognitive impairment in APP/PS1 transgenic mice, Aβ_1-42_ brain perfusion mice and 3 × Tg-AD transgenic mice. However, clinical studies (NCT01297218, NCT01696591) have not demonstrated the beneficial effects of exogenous stem cell therapy in AD [205, 206].

#### Altered intercellular communication

Ageing is related to gradual changes in cell–cell communication, such as defects in neural, neuroendocrine and hormonal signalling pathways, which impair homeostasis and hormone regulation [207].

Alterations in neurotransmitters are closely related to synaptic alterations and neuronal loss in AD. Studies employing single-cell sequencing (snRNA-Seq) have revealed molecular pathways that regulate neuronal dysfunction [208] and demonstrated that excitatory neurons from AD patients show dysregulated expression of genes that are involved in neurotransmitter release, synaptic vesicular circulation and glutamate metabolism. Several differentially expressed genes are involved in postsynaptic scaffold formation, glutamate receptor trafficking and calmodulin signalling [209]. AD is associated with a decreased number of excitatory neurons and downregulation of *NTNG1*, a gene that regulates neurite development [210]. As secretory cells of the CNS, astrocytes release neurotransmitters (e.g. glutamate or GABA), neuromodulators (e.g. renal acid) and trophic factors. Some studies employing snRNA-Seq have indicated that genes related to glutamate receptor subunits (e.g., *GRIA2*, *GRM3* and *GRID2*) are altered in astrocytes of AD patients [168, 211]. The metabolic reprograming of astrocytes in AD may affect their capacity to maintain nervous system homeostasis. Astrocytes may degrade harmful fatty acids (FAs) generated by hyperactive neurons. *APOE4*, a gene associated with the risk of AD, impairs lipid balance and energy transmission in the brain. It affects neuronal function by decreasing the separation of FAs from lipid droplets, the efficiency of FA transport and the capacity of astrocytes to degrade FAs, thereby impairing the metabolic and synaptic support for neurons [212]. Neurons and astrocytes closely interact with each other throughout the neurotransmitter cycle, also known as the glutamate/GABA-glutamine cycle. The metabolic abnormalities in astrocytes during the development of AD lead to defective metabolic support for neurons, resulting in synaptic dysfunction and neurodegeneration [213].

In addition, impaired abilities of microglia to detect and regulate neuronal activity may contribute to neuronal dysfunction in patients with AD. The ability of microglia to regulate neuronal activity may be compromised by changes in their metabolic state, which are partly associated with cellular stress and glycolysis. Ageing is often associated with an increased amount of inflammatory cytokines in the body; however, whether there are age-related alterations in cytokine signalling pathways and intercellular communication remains controversial. Xu et al. [214] reported that intracerebroventricular injection of tumor necrosis factor (TNF) α or interferon-γ increases the number of transcripts of CXCL9 and ICAM-1 (intercellular adhesion molecule-1) in both young and old mice. Additionally, the proportions of CD3^+^, CD4^+^ and CD8^+^ T lymphocytes in the brain parenchyma and choroid plexus increase with advancing age. These results suggest that the exposure of the brain to inflammation can lead to age-related lesions and the responses to inflammation become more severe with ageing.

The macrophage populations activated in response to brain ageing include parenchymal microglia, boundary-associated macrophages and mononuclear cells. Silvin et al. demonstrated that the embryonically derived TREM2-dependent disease-associated microglia (DAM) exert neuroprotective effects, whereas the monocyte-derived TREM2-expressing disease inflammatory macrophages (DIM) play a pro-inflammatory role. These two cell types accumulate in the brain during ageing [215]. Fahrenhold et al. [216] performed immunostaining on post-mortem brain tissues and found that TREM2 was not expressed in microglia but appeared to be a marker of monocyte recruitment in the brain. This finding suggests that TREM2 is a marker of recruited peripheral immune cells in the brain. Additionally, single-cell RNA sequencing revealed that in addition to neuronal ageing phenotypes, transcriptome changes in endothelial cells and microglia were prevalent in the ageing brain, whereas T-cell infiltration was prevalent in old neurogenic niches [199]. T cells may suppress the proliferation of NSCs by secreting interferon-γ both in vitro (co-culture) and in vivo. Therefore, T cells and NSCs can interact in the ageing brain and serve as potential targets for counteracting age-related impairments in brain function.

The BBB controls the homeostasis of the brain microenvironment by regulating the exchange of molecules and cells between blood and the brain [217]. The effect of vascular status on the BBB is a significant risk factor for AD [218]. Vascular disruption occurs in the early stage of AD and may affect cognitive impairment. Disruption of the BBB is related to endothelial and pericellular degeneration as well as changes in the basement membrane and astrocyte end-feet. BBB destruction allows the infiltration of blood-derived cells and circulatory factors into the brain and prevents Aβ clearance [219]. Microglia associated with capillaries control blood flow through purinergic actions. In AD, microglia may release factors that impair the integrity of endothelial tight junctions. Chemokines released by microglia may affect the inflamed condition of the endothelium membrane, which may, in turn, lead to neutrophil adhesion and reduced blood flow owing to capillary stalling [220]. CD19 is expressed in brain mural cells surrounding the endothelium, which are necessary for maintaining the integrity of the BBB. Parker et al. [221] evaluated CD19 expression in brain mural cells and verified perivascular presence of CD19 protein in human brain. Experiments in mice showed that although mice had less abundant CD19^+^ mural cells in the brain, CD19-directed therapies for tumors led to BBB leakiness, which was indicative of neurotoxicity. These findings suggest that CD19 may be an effective strategic target for improving the integrity of the BBB.

Similarly, impaired meningeal lymphatic drainage has been found to exacerbate the inflammatory response of microglia in AD [222]. Meningeal lymphatics are channels in the brain and transport large molecules, including Aβ, from cerebrospinal and interstitial fluids, whereas other metabolic wastes are cleared through the vascular bypass [223]. Under healthy conditions, immune cells usually cannot enter the meninges of the brain; therefore, meningeal lymphatic vessels transport macromolecular metabolites to the lymph nodes of the neck, where they are cleared by immune cells. However, the metabolic wastes will rapidly accumulate again [224]. Wu et al. [225] demonstrated that borneol accelerated the lymphatic clearance of Aβ by improving meningeal lymphatic drainage and alleviated cognitive impairment in Aβ-injected rats. This finding suggests that the cognitive ability in AD is related to the ability of meningeal lymphatic vessels to clear macromolecular metabolic wastes. Therefore, repairing and increasing the function of meningeal lymphatic vessels may be an effective strategy for AD prevention and treatment.

In conclusion, some researchers have attempted to identify cell–cell communication networks that may delay ageing and prolong survival [226]. Identifying commonly altered pathways may facilitate the development of novel therapeutic approaches involving numerous cell types [227]. In AD, almost all cell types including astrocytes, microglia, and neurons have immunological responses, including transcriptional responses involving cytokines, chemokines and MHC signalling pathways. Synaptic plasticity and unfolded protein response may be associated with the MHC signalling pathway [228]. Consequently, regulating the communication networks between different cell types is a promising strategy for treating neurodegenerative diseases such as AD (Fig. 2).

### Systemic hallmarks

#### Chronic inflammation

Many studies have demonstrated that patients with AD have a chronic neuroinflammatory response. On the one hand, the neuroinflammatory response stimulates microglia to phagocytose Aβ, thereby decreasing Aβ accumulation. On the other hand, severe inflammation may cause irreparable nerve tissue damage [105]. Uyar et al. [232] conducted a large-scale integrated study based on single-cell analysis and found that the expression of genes related to cellular inflammation is usually upregulated with ageing. Although Aβ deposition is a primary cause of AD, several genetic variations in the inflammatory pathway may synergistically influence the risk of AD [233]. Dysregulation of inflammation during ageing may be an important cause of the progression of AD. In the CNS, microglia are activated after they detect ‘danger’ signals (e.g., Aβ). Upon activation, they produce pro-inflammatory factors and recruit monocytes and dendritic cells to the brain via disrupted BBB or the lymphatic system of the brain [234]. Sustained activation of microglia may lead to persistent inflammation and underlie the central neuropathophysiology of age-related neurodegeneration and cognitive decline. [235]. Cole et al. [236] found that APOBEC1-induced RNA editing was key to maintaining the resting state of microglia. Similar as bone marrow-derived macrophages, RNA editing in microglia alters the abundance of edited proteins that coordinate multiple cellular pathways. In contrast, mice lacking APOBEC1 in microglia showed abnormal regulation, manifesting as aggregation of activated microglia, abnormal myelination, elevated inflammation and lysosomal abnormalities, which led to behavioural and psychological deficits. Another study revealed that compared with brain tissues of young mice, the brain tissues of old mice showed over-recruitment of peripheral CCR2^+^ macrophages and augmented inflammatory responses after traumatic brain injury [237]. Baik et al. [238] found that chronic Aβ treatment induces innate immune tolerance in microglia, due to the defects in cellular metabolism. They also demonstrated that recombinant interferon-γ rescues the glycolytic metabolism and immune functions of microglia and alleviates the symptoms of AD in transgenic mice.

**Fig. 3:**
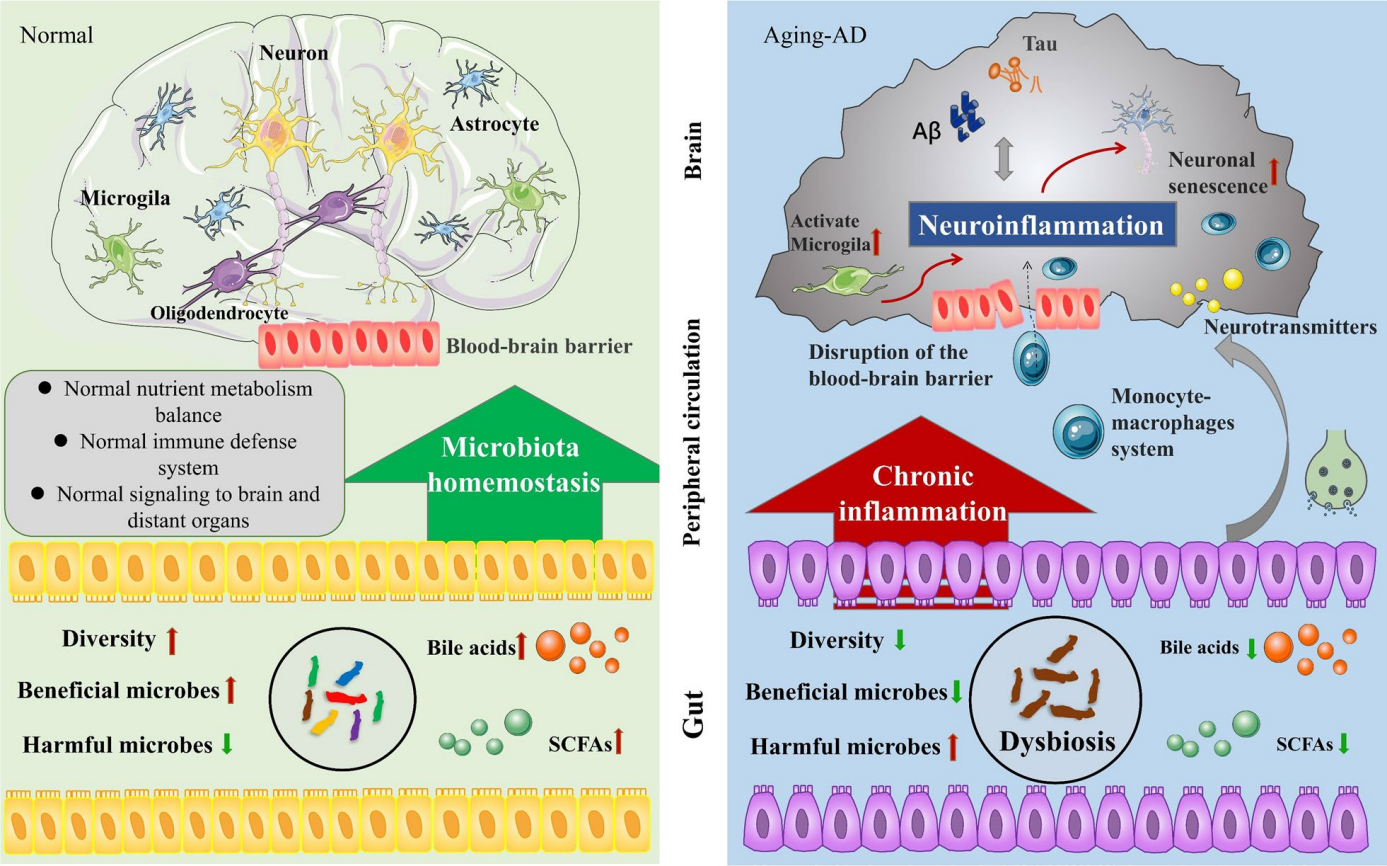
Changes in systemic ageing markers between normal and ageing (in AD) brains. Microbiota homeostasis maintains normal nutrient metabolism, immune defense and signaling transduction to the brain and other organs [229]. Dysbiosis, including decreased diversity of microbiota, decreased abundance of beneficial microbes, increased abundance of harmful microbes and decreased levels of bile acids and SCFAs, can cause a persistent inflammatory reaction [230]. Moreover, disruption of the BBB allows macrophages to enter the brain and aggravate neuroinflammation [231]. *AD* Alzheimer’s disease, *SCFAs* short-chain fatty acids

In conclusion, in AD, neuroinflammation is caused by aggregation of pathogenic tau proteins through activation of microglia and astrocytes, and the chronic inflammatory state is responsible for the subsequent neuronal loss (Fig. 3). However, anti-inflammatory drugs do not mitigate or prevent the development of AD [239]. Chronic inflammation may be a result of persistent pathophysiology in AD, which may in turn worsen the symptoms. Neuroinflammation, primarily induced by microglia and astrocytes, usually leads to dysfunction and degeneration of adjacent neurons, as the activated glial cells have reduced ability to maintain and support neurons while having increased expression of pro-inflammatory cytokines. This phenomenon is similar to ageing, in which the accumulation of senescent cells increases and initiates primary senescence, disrupting the ability of cells to maintain homeostasis and increasing their susceptibility to diseases. With disease onset, a second episode of ageing is triggered, resulting in a vicious feedback loop. Inflammation and ageing may worsen this feedback loop. Therefore, regulating the development and progression of chronic inflammation is a feasible preventive strategy for age-related diseases.

#### Dysbiosis

Disruption of the bidirectional bacteria–host relationship results in ecological disequilibrium. The microbiome pattern of healthy ageing in a majority of individuals is defined by the loss of key groups such as *Bacteroides*. *Alistipes putredinis* may minimize the risk of pathogenic infections and promote intestinal homeostasis, thereby decreasing the susceptibility to age-related chronic illnesses [240].

The human microbiome comprises bacteria found in the intestinal tract, oral cavity, nasal cavity and other organs interacting with the external environment. An imbalance of the microbiome in any organ may cause systemic abnormalities, with the intestinal microbiome being the most influential. The composition of intestinal microbes can be influenced by the increased intestinal barrier permeability and immune cell activation [241]. Enhanced secretion of lipopolysaccharides and bacterial amyloid proteins caused by gut dysbiosis may trigger activation of brain-resident immune cells, leading to impairments of intestinal permeability and BBB function, neuroinflammation, neuronal loss and eventually AD [242]. By cleaving Aβ precursor protein and tau proteins in the brain, the CCAAT/enhancer-binding protein/asparagine endopeptidase (C/EPP/AEP) signalling pathway contributes to the AD pathologies. Chen et al. [243] found that the gut dysbiosis in AD mice increases with age and is related to the C/EBP-β/AEP pathway. Compared with the microbiota of aged wild-type mice, the microbiota of aged AD mice accelerate the symptoms of AD in young AD mice, through the active C/EBP- β/AEP signalling. Antibiotic treatment and microbiome transplantation [244] can reduce the activity of this signalling pathway and improve cognitive function. Wang et al. [245] found that in AD mice, with the progression of AD, alterations in the gut microbiota composition result in the peripheral accumulation of phenylalanine and isoleucine, thereby promoting the differentiation and proliferation of pro-inflammatory T helper 1 (Th1) cells. These changes were also found in two cohorts of patients with mild cognitive impairment due to AD. These results suggest that changes in the gut microbiota composition play a role in early stages of AD.

In addition to gut microbiota, oral and nasal microbiota have also been found to be involved in the development of AD [246]. Microbes shared by intestinal, oral and nasal microbiota, particularly *Porphyromonas gingivalis*, may be associated with AD through a common pathway in the innate immune system [247]. Dominy et al. [248] found that oral *P. gingivalis* infection in mice led to bacterial colonisation in the brain. Furthermore, increased levels of Aβ_1-42_ and gingival protease (a toxic protease) are observed in the brains of patients with AD, indicating that these two factors are associated with tau- and ubiquitin-associated pathological changes. AD seems to involve microbiome in the brain [249]. The dominant microorganisms consistently detected in brain tissues from AD patients or animal models are *Spirochaeta*, herpes simplex virus, *Chlamydia pneumoniae* and *Porphyria gingivalis* [250]. These microorganisms are capable of altering the opsonophagocytosis activity of microglia and exacerbating AD disease [251].

In conclusion, dysbiosis can lead to enhanced secretion of lipopolysaccharides and amyloid protein, which may disrupt intestinal and BBB permeability. In patients with cognitive impairment, inflammatory microbes in the gut microbiota are associated with peripheral inflammation and Aβ deposition in the brain. Oral microbiome may influence the risk of AD through circulatory or neural pathways in the brain. Combined antibiotic therapy has been demonstrated to be effective in animal models of AD, highlighting the important role of dysbiosis in AD [252]. Remodelling the microbiota homeostasis may be a novel therapeutic strategy for AD.

## Novel techniques to study the mechanisms underlying ageing in AD

As ageing is an inevitable process, elucidating the mechanisms underlying ageing is of great significance to the field of life sciences. With the continuous development of bioinformatic techniques, several novel methods have been used to understand these mechanisms [253].

### Mendelian randomization (MR)

Given the complexity of factors affecting ageing, assessing the relationship between these factors and age-associated diseases is necessary for developing effective therapeutic strategies. Mediation analysis is a traditional approach to elucidating the effects of exposure on an outcome. However, traditional mediation analysis with non-instrumental variables is limited by several methodological difficulties, including bias due to the confounding between an exposure, mediator and outcome, and measurement errors. MR is a causal inference method that examines the influence of biological factors on diseases using genetic variants associated with the factors as instrumental variables [254]. MR uses the published genetic data to explore the causal relationship between exposure and outcome. Similar to randomised controlled trials (RCTs), MR ‘randomises’ the participating genes based on one or more alleles that influence risk factors to determine whether carriers of these genetic variants have a different risk of developing the disease compared with non-carriers. On the contrary, traditional observational studies examine the effects of exposure through questionnaires, biochemical markers or imaging approaches. Genetic variants are present at birth and remain stable throughout life. To date, numerous genetic variants strongly associated with specific traits have been identified, and many large-sample genome-wide association studies (GWAS) have published pooled data on several exposures and disease-related genetic variants. These aggregated data enable researchers to estimate genetic associations in large-sample studies, thereby facilitating the development of MR. As MR can avoid the influence of residual confounding factors on the accuracy of the results, the associations identified via MR are more reliable than those reported in observational studies or RCTs. MR can be used to improve the causal inference in mediation analysis using alleles that follow random assignment without being affected by confounding factors and not subject to reverse causation (which is observed in traditional epidemiological studies) [254]. For example, telomere shortening is difficult to detect in a population; however, MR translates it into a detectable mutation [255]. A MR study by Rodríguez et al. showed that a lower telomere length is causally related to an increased risk of AD [256]. To date, studies have neither reported a proven direct cause of AD nor revealed any risk factors that are easily modifiable, resulting in the lack of effective therapeutic strategies. MR method may remarkably improve the identification of risk factors for AD and facilitate the development of effective therapeutic strategies.

### Omics analysis

The omics technology has revolutionized the detection of genes (genome), mRNAs (transcriptome), proteins (proteome) and metabolites (metabolomics) in cells.

#### Single-cell sequencing

Traditional studies usually focus on animal (in vivo) or cell (in vitro) models without consideration of the heterogeneity and the interactions among different cell types. Single-cell sequencing, an emerging bioinformatic technology, allows the analysis of heterogeneity among cells and cell–cell interactions [28, 257]. At present, a complete set of single-cell and spatial multiomics technologies is available, including single-cell transcriptome sequencing (sc-seq), single-cell ATAC sequencing (sc-ATAC-seq), single-cell immune library sequencing (scTCR/BCR-seq) and spatial transcriptomic sequencing. Compared with conventional omics techniques, single-cell sequencing allows independent analysis at the cellular level to assess heterogeneity among different cell types. The subsequent development of spatial transcriptomic sequencing allows direct mapping of the functions of cells in different parts of the body [258]. In studies of CNS diseases, single-cell sequencing has been primarily used to examine changes in the gene expression profiles in neurons, glia, microglia and immune cells to elucidate the pathogenesis and explore new therapeutic targets. To date, in studies of AD, single-cell sequencing has been utilized mainly for the following purposes: constructing a brain atlas; examining cells in the hippocampus, cortex and organoids; assessing the risks of diseases and finding novel treatment modalities (Table 1). In a previous study, transcriptomics analysis of different cell subsets in the PFC of 48 AD patients revealed AD pathology-related cell subsets characterized by regulators of myelination, inflammation and neuronal survival, revealing the molecular characteristics and pathogenesis of AD [259]. In another study, single-cell sequencing in blood and cerebrospinal fluid samples from AD patients revealed changes in peripheral and central acquired immune responses in the patients, suggesting changes in the immune microenvironment of AD [260]. Similarly, ncRNA sequencing may help identify AD-specific regulatory ncRNAs, thus providing a theoretical basis for considering ncRNAs as drug targets [261]. In addition, single-cell transcriptomic sequencing studies have demonstrated that microglia, interneurons and endothelial cells have differentially expressed genes in AD [259, 262]. Disruption of signalling pathways directly associated with these marker genes, such as inhibition of fractal and purine signalling pathways in microglia, and inhibition of neuropeptide signalling pathways in interneurons, may affect neural circuits. The loss of marker genes indicates the loss of transcription programs that control the identity of different cell types in AD, which may lead to cell senescence. Miller et al. [28] analyzed the single-cell sequencing data of neurons isolated from the PFC and the hippocampus of patients with AD and healthy individuals. They found that patients with AD had more somatic DNA changes and molecular patterns that differed from the age-related patterns of mutation observed in normal neurons. Additionally, AD was found with increased nucleotide oxidation associated with abnormal protein homeostasis. These findings provide valuable insights into the sequence of molecular and cellular processes that occur throughout the progression of AD [263].

**Table 1:** Summary of recent studies on AD utilizing single-cell RNA sequencing

Study and year	Platform	Region	Data accessibility	Main findings
Keren-Shaul et al. 2017 [264]	Illumina NextSeq 500 sequencer	Whole brains of 5xFAD mice and C57/BL6 mice	GEO: GSE98969 (single-cell RNA-seq) and GEO: GSE98970 (iChIP)	A novel microglial type associated with neurodegenerative diseases was described. The markers, spatial localisation and pathways associated with this cell type were identified
Mathys et al. 2017 [265]	Illumina HiSeq 2000 platform	Hippocampus of AD mice and control mice	GEO: GSE103334	Two molecularly distinct phenotypes of reactive microglia were identified, characterized by modules of co-regulated type I and type II interferon response genes, respectively. The study also identified heterogeneity in microglial responses to neurodegeneration, disease stage-specific microglial states, cell reprogramming trajectory of microglial response to neurodegeneration and the underlying transcriptional pathways
Mathys et al. 2019 [259]	10× Genomics platform	Prefrontal cortex of patients with AD and healthy individuals	https://www.radc.rush.edu/docs/omics.htm (snRNA-seq PFC)	The study identified different transcriptomic subsets, including those associated with pathology, characterised by regulators of myelination, inflammation and neuronal survival. The strongest disease-related changes occur early in the progression of AD and are highly cell type-specific, whereas in the later stages of AD, different cell types show similar upregulation of genes, mainly those involved in stress responses
Cosacak et al. 2019 [266]	10× Genomics platform	Zebrafish brain	GEO: GSE118577	The study provided extensive data on the molecular basis of NSC plasticity in the brains of adult zebrafish models of AD
Gate et al. 2020 [260]	Illumina HiSeq 4000 platform	Peripheral blood mononuclear cells of 97 healthy individuals, 31 patients with MCI, 28 patients with AD and 8 patients with PD	GEO: GSE134578	The immune-related features of AD included an increase in the number of CD8^+^ T effector memory CD45RA^+^ (TEMRA) cells, and the proportion of CD8^+^ TEMRA cells was negatively associated with cognitive function. In addition, single-cell RNA sequencing showed that T cell receptor signalling was enhanced in these cells. The findings revealed adaptive immune responses in the blood and cerebrospinal fluid of patients with AD and provided evidence that cloned, antigen-experienced T cells patrol the intracranial space of the brain affected by age-related neurodegenerative diseases
Zhong et al. 2020 [267]	Illumina Hiseq-PE150 platform	Hippocampus of APP23 mice	GEO: GSE141044	Comparative transcription analysis revealed various changes in different subtypes of hippocampal neurons in APP23 mice compared with control mice, and validated transcriptional changes in these neurons during disease progression
Xu et al. 2020 [268]	Illumina NovaSeq 6000 platform	Peripheral blood mononuclear cells of amyloid-positive AD patients and amyloid-negative healthy individuals	GEO: GSE181279	Five immune cell subsets were revealed: CD4^+^ T cells, CD8^+^ T cells, B cells, natural killer cells and mononuclear macrophages. The characteristic changes in the proportions of these cell subsets and their gene expression patterns in AD were revealed. Protein–protein interaction network and pathway enrichment analyses revealed 31 cell type-specific key genes, including abundant human leukocyte antigen genes and multiple immune-related pathways. The study also revealed high-frequency amplification clones of T and B cells and decreased T cell diversity in AD. As clonal amplification suggests the activation of adaptive immune responses to specific antigens, the finding suggests that peripheral adaptive immune responses, especially those mediated by T cells, may play a role in the pathogenesis of AD
Leng et al. 2020 [269]	10× Genomics platform	Post-mortem brain tissues	GEO: GSE147528	The study identified a subpopulation of reactive astrocytes characterized by reduced expression of genes involved in homeostasis
Walgrave et al. 2021 [261]	10× Genomics platform	Human hippocampal and serum samples	GEO: GSE172402	The study revealed that miR-132 is one of the most consistently down-regulated miRNAs in AD and an effective regulator of adult hippocampal neurogenesis (AHN), and plays a cell-autonomic neurogenic role in adult neural stem cells and their progeny. AHN was shown to be directly affected by AD pathology. miR-132 replacement in the hippocampus of adult AD mice restored AHN and alleviated associated memory deficits
Lee et al. 2021 [270]	Illumina HiSeq4000 platform	Hippocampus of AD mice model	Cohort I (GSE160512), cohort II (GSE181786) and cohort III (GSE153895)	The spatial distribution of two types of oligodendrocytes with distinct transcriptional states was revealed. TREM2-deficient animals exhibited drastically delayed microglial responses to tau and Aβ pathology, whereas non-microglial (oligodendrocytes, astrocytes and T cells) responses remained unaltered
Freitag et al. 2022 [271]	10× Genomics platform	Cerebral hemisphere of APP/PS1 mice	GEO: GSE206202	The study identified a population of microglia characterized by elevated AXL levels and expression of phagocytosis- and cell-migration-related genes. Subsequent proteomic analysis of microglia isolated from APP/PS1 mice validated the anti-inflammatory and cytoskeletal effects of spermidine. Autophagy induced by spermidine altered the TLR3- and TLR4-mediated inflammatory processes, Aβ phagocytosis and motility in primary microglia and astrocytes
Lampinen et al. 2022 [272]	Illumina NovaSeq S1 platform	Olefin mucosa cells extracted from three cognitively healthy individuals (mean age, 71.7 years) and five patients with AD (mean age, 67.2 years)	European Genome-phenome Archive (EGA, https://ega-archive.org/, 10 February 2022) under the accession ID EGAS00001006019	Single-cell RNA sequencing revealed altered expression of mitochondrial localization-related genes in olefin mucosa cells in AD. This finding was corroborated by functional assays, which revealed altered mitochondrial respiration and decreased ATP generation
Wood et al. 2022 [273]	Illumina NextSeq 2000 platform	Cerebral hemisphere of APP^NL−F/NL−F^ knockin mice	All data reported in this study will be shared by the corresponding author upon request	The TREM2-dependent genes are all involved in phagocytosis and degradation. The decrease in phagocytosis markers is associated with an increase of tiny plaques in mice with TREM2 mutations. Furthermore, in the presence of R47H mutation that prevents the increase in TREM2 expression, there were still small increases in TREM2 protein and microglial density on plaques. The results indicate that the interaction between microglia and plaques and the activity of TREM2 are both required for microglia to respond appropriately to amyloid-related diseases

Single-cell sequencing has advanced our understanding of diverse cell types in the brain, including oligodendrocytes, microglia and neurons. Comparing the sequencing data between humans and mice or non-human vertebrates can reveal markers of conserved or specific cell types, including the proportions of different cell types in tissues and species-specific gene expression patterns. Single-cell sequencing can be further improved by developing methods for long-term storage of tissues and cells without damage to genes, more standardized data quality control methods, dimensionality reduction in data analysis, and increasing the reliability of statistical analysis. At present, studies employing single-cell sequencing for examining brain diseases are lacking. It is necessary to understand the interactions between various types of cells during disease development. Additionally, AD-specific transcriptional abnormalities may not represent biological differences, as the changes in gene transcripts may not transmit into protein level changes. In addition, non-genomic processes that may not be observed via single-cell profiling, are also involved in the dysfunction in AD, including post-translational modifications and mRNA regulatory processes involved in local protein synthesis in various cell compartments and types. Altogether, single-cell sequencing enables comprehensive understanding of the pathophysiology of AD from perspectives of different cell types and multi-omics features, and would facilitate the development of novel strategies for preventing and treating AD.

#### Spatial transcriptomics

Molecular mapping techniques such as genomics and proteomics involve tissue separation, which results in the loss of morphological and spatial information. Recent advances in spatial molecular mapping methods have facilitated thorough molecular characterisation of cells while preserving their spatial and morphological integrity [274]. As compartmentalization is a fundamental feature of the brain, understanding the integrative functioning of different parts (in brain tissue) is crucial. Single-cell analysis helps understand this coordinated functioning, and spatial transcriptomics analysis provides insights into the whole-brain transcriptomic pattern associated with the pathological features of AD. In particular, spatial transcriptomic analysis is advantageous in ageing-related studies. Single-cell RNA sequencing requires the generation of single-cell suspensions, which are difficult to produce in older mice. Visium Spatial Gene Expression, a spatial transcriptomics technique, uses tissue slices as the input, overcoming the aforementioned limitation [275]. Studies employing pathological and immunohistochemical analyses have demonstrated the presence of complex inflammation-like changes around aggregated Aβ in patients with AD; however, the molecular changes and cellular interactions that characterize this response were not clear. Chen et al. [276] used the spatial transcriptomics technique and discovered transcriptional changes in tissue domains within a 100-μm diameter surrounding aggregated Aβ. They also demonstrated early changes in the co-expression network of myelin-related genes and oligodendrocyte genes. With technological advancements, the obstacles to spatial transcriptomics technique, including resolution and sensitivity restrictions, and feasibility of different tissues, are gradually improved. The spatial transcriptomics technique is compatible with paraffin-embedded tissues and allows retrospective analysis of samples collected over decades. Future advancements may facilitate the systematic assessment of larger tissues to generate 3D organ or organism-level maps and observe changes in gene expression throughout the transcriptome over time [277]. In addition to overcoming these technological obstacles, innovative computer tools and analytical methods should be developed in the future. These innovations may improve data analysis to elucidate anatomical patterns (essential characteristics of spatial transcriptomic datasets) and the underlying molecular mechanisms of genes.

#### Spatial proteomics

The function of a protein varies across different intracellular locations. Therefore, understanding the subcellular localization of a protein is important for determining its function. Upon synthesis, proteins function effectively only after being transported to the correct organelles. Spatial proteomics refers to the mapping of proteins at the single-cell or the tissue level. This technique allows detection of changes in the spatial expression profiles of proteins in diseases, providing a novel strategy for identifying biomarkers and developing effective diagnostic and therapeutic methods [278].

Prokop et al. used NanoString digital spatial profiling (DSP) to analyze the protein expression profiles of microglia in different brain regions and showed differences in immune response patterns across the examined brain regions [279]. DSP is an emerging technique that allows spatial analysis of proteins across multiple regions of interest in formalin-fixed paraffin-embedded tissue sections [280]. Walker et al. used DPS to analyze the protein expression profiles of NFT-bearing neurons, non-NFT-bearing neurons, and their neuronal microenvironment in the hippocampus. The results showed that low energetic and oxidative stress levels are important for the maintenance of neurons and their synaptic connections [281]. Vijayaragavan et al. developed a nanoscale framework integrating spatial proteomics and multiplexed ion beam imaging. This framework was used to examine neuropathological features, identify cell types and protein lesions in the hippocampus during different stages of AD, and assess the direct relationship between microglia and tau-related pathological changes in the CA1 hippocampal region in patients with AD [282]. In another study, the quantitative multiplex immunohistochemistry and visual colourimetric staining to enhance regional protein localisation (QUIVER) technique was developed to overcome the problem of unclear postmortem tissue immunostaining. Five molecular phenotypes of microglia/macrophage were identified in patients with AD using QUIVER in conjunction with digital pathological tools. The reactive and homeostatic microglial/macrophage phenotypes exhibit spatial polarization toward and away from Aβ, respectively. These findings suggest that QUIVER is a valuable tool for examining pathological changes in the brains of older adults [283]. Spatial proteomics is of great significance in clinical diagnosis and treatment. DISCO-MS, a technique that combines ultra-sensitive mass spectrometry (MS) with whole-organ/whole-organism clearance and imaging, allows unbiased proteomic analysis of preclinical and clinical tissues to identify diagnostic and therapeutic opportunities for complex diseases [284].

The development of single-cell proteomics is slower than that of genomic technology partly because that the proteomes cannot be amplified like nucleic acids and hence require extremely sensitive detection instruments. The current spatial proteomics methods used for characterizing protein expression have limitations. For example, the multi-immunofluorescence imaging coverage is usually limited to 20–100 proteins, and methods such as MS are not sensitive enough to capture the proteome at high spatial resolution [285]. The high resolution of modern microscopes and the increasing sensitivity of MS have increased the accuracy of proteomic analysis in natural subcellular settings; however, using images to measure protein abundance at the single-cell level remains a key challenge. Therefore, future studies on spatial proteomics should integrate AI-driven image analysis with automated single-cell or single-core laser microdissection with ultra-sensitive MS [286].

#### Spatial metabolomics

Spatial metabolomics that combines imaging mass spectrometry with metabolomic techniques examines the spatial molecular profiles in tissues in an unlabelled and untargeted manner. In the field of medicine, the spatial metabolomic technology can be used to localize molecular changes in tissue sections. Understanding the spatial localisation of metabolites is particularly important for addressing the complexity and heterogeneity of neurodegenerative diseases and their microenvironments. A spatial metabolomics study by Wang et al. [287] revealed effects of changes in gut microbiota composition on dopamine in the striatum of mice. Disturbance of microbial homeostasis is an important factor in AD. The spatial organization of metabolic processes of the microbial community, the interactions among microbes, and the exchange of metabolites between cells in various locations, can influence the overall metabolic activity of the community. In a recent review, Dal et al. [288] have discussed the mechanisms that drive the spatial arrangement of metabolic processes from the perspective of spatial metabolome and emphasized the influence of spatial organization of metabolic processes on the ecology and evolution of microbial communities. Although the spatial metabolomic technology holds great promise in studies on disease mechanisms, tissue classification, biomarker discovery and drug development, further development is required to improve the annotation of metabolites and the accuracy of spatial algorithms [289].

#### Multiomics analysis

AD is a complex multifactorial disease with changes in the multilayered biological networks, including protein imbalance (Aβ and tau aggregation), neuroinflammation and abnormal metabolism. Omics such as genomics, transcriptomics, proteomics and metabolomics are embedded within the theoretical and computational framework and can generate interpretable readings that describe the entire biological continuum of diseases from a systemic view [290, 291]. The combined use of multiple omics analyses can facilitate the identification of reliable biomarkers and improve clinical management of AD [292]. Extracellular vesicles (EVs), which are lipid bilayer particles released by most cells, play a crucial role in intercellular communication and systemic diseases. A multi-omics study conducted by Cohn et al. [293] was the first to report that many genetic risk factors of AD are associated with microglia-specific intercellular communications. The study revealed that approximately 1000 AD-specific proteins, 600 lipids and 100 miRNAs are present in microglia-derived EVs. In addition, the protein and lipid transports were found to be abnormal in AD, accompanied by a proinflammatory lipid profile, dysfunctional endolysosomes and upregulation of four miRNAs related to immune and cellular senescence signaling pathways in AD. Therefore, the analysis of microglia-derived EVs allows for development of new EV-related biomarkers and provides a framework for future larger-scale multi-omics studies on patient-derived cell type-specific EVs. Integration of multi-omics data into a synthetic neural network may help detect and map upstream mechanisms underlying the pathological changes in AD and downstream molecular effects in preclinical settings [294].

## Potential therapeutic targets

Given that suppressing ageing can extend life expectancy and delay the development of age-related disorders, anti-ageing strategies developed based on biological, chemical, nanotechnological and immunological principles have attracted much attention. As of January 2022, 143 anti-AD drugs were under investigation in trials in the US. Of them, 119 (83.2%) drugs account for disease-modifying therapies, whereas only 24 (16.8%) drugs account for symptomatic treatment [295].

Anti-ageing efficacy is an important focus of studies on anti-AD drugs (Table 2). Among the mechanisms of action, proteostasis has been extensively studied [296]. A reason for the increased focus on proteostasis is that AD is characterized by abnormal protein deposition long before its association with ageing [297]. Immunotherapies offer the possibility of targeted elimination of abnormal proteins. A total of 12 monoclonal antibodies (mAbs) against Aβ have been developed for AD treatment, most of which are undergoing clinical trials. A new direction of mAb research is to target more-specific Aβ types and improve therapeutic effects while reducing side effects. Lecanemab, a newly developed mAb against Aβ [298], has a high affinity for large, soluble Aβ protofibrils and is currently undergoing investigation in a phase III trial [299]. Additionally, TRx0237 has entered a phase III clinical trial as a tau-aggregation inhibitor [300]. Liu et al. isolated homologous prostrong mycin from the marine bacterium *Streptomyces tendae* MCCC 1A01534. This compound inhibited tau aggregation to protect HT22 cells and exhibited low cytotoxicity, demonstrating its therapeutic potential for AD [301]. Furthermore, regulation of nutrient sensing can alleviate the symptoms of AD. The insulin sensitizer metformin can significantly decrease the risk of AD [302] and has demonstrated therapeutic efficacy in animal models of AD [303]. Metformin is currently undergoing investigation in a phase III clinical trial [304].

**Table 2:** Anti-AD drugs with anti-ageing properties in trials

Candidate drugs	Relevant AD hallmark of target	Mechanism of action	Outcome	Current clinical trial
Lecanemab	Loss of proteostasis	Monoclonal antibody directed at Aβ plaques and oligomers	Reduces Aβ deposition	Phase 3 (NCT03887455)
Aducanumab	Loss of proteostasis	Monoclonal antibody directed at Aβ plaques and oligomers	Reduces Aβ deposition and controls inflammation	Phase 3 (NCT04241068)
Donanemab	Loss of proteostasis	Monoclonal antibody specific for the pyroglutamate form of Aβ	Reduces Aβ deposition	Phase 3 (NCT04437511)
Donanemab & Aducanumab	Loss of proteostasis	Combination of two monoclonal antibodies	Reduces Aβ deposition	Phase 3 (NCT05108922)
Simufilam (PTI-125)	Loss of proteostasis	Filamin A protein inhibitor	Stabilizes the interaction between amyloid-alpha-7 and nicotinic receptor	Phase 3 (NCT04994483)
Gantenerumab	Loss of proteostasis	Monoclonal antibody directed at Aβ plaques and oligomers	Reduces Aβ deposition	Phase 3 (NCT03444870)
TRx0237	Loss of proteostasis	Inhibitor of tau protein aggregation	Reduces tau protein aggregation	Phase 3 (NCT04770220)
Valiltramiprosate (ALZ-801)	Loss of proteostasis	Prodrug of tramiprosate	Inhibits the aggregation of Aβ into toxic oligomers	Phase 3 (NCT04770220)
ACI-35	Loss of proteostasis	Active immunotherapy targeting tau	Reduces tau protein aggregation	Phase 2 (NCT04445831)
Nilotinib BE	Disabled macroautophagy	Tyrosine kinase inhibitor; autophagy enhancer	Increases clearance of Aβ and tau	Phase 3 (NCT05143528)
Losartan and Amlodipine and Atorvastatin + exercise	Deregulated nutrient sensing	Angiotensin II receptor blocker (losartan), calcium channel blocker (amlodipine) and cholesterol agent (atorvastatin)	Regulates vascular energy metabolism	Phase 3 (NCT02913664)
Metformin	Deregulated nutrient sensing	Insulin sensitizer	Improves glucose metabolism in the CNS	Phase 3 (NCT04098666)
Semaglutide	Deregulated nutrient sensing	GLP-1 agonist	Reduces neuroinflammation and improves insulin signalling in the brain	Phase 3 (NCT04777396)
Tricaprilin	Mitochondrial dysfunction	Caprylic triglyceride	Induces ketosis and improves mitochondrial and neuronal function	Phase 3 (NCT03446001)
MitoQ	Mitochondrial dysfunction	Mitochondria‐targeting antioxidant	Reduces systemic oxidative stress and increases cerebral oxygenation	Phase 1 (NCT03514875)
AGB101 (low-dose levetiracetam)	Cellular senescence	Synaptic plasticity/neuroprotection	Reduces Aβ-induced neuronal overactivity	Phase 3 (NCT03486938)
Atuzaginstat (COR388)	Cellular senescence	Synaptic plasticity/neuroprotection	Reduces Aβ-induced neuronal overactivity	Phase 3 (NCT03823404)
Dasatinib + Quercetin	Cellular senescence	Tyrosine kinase inhibitor (dasatinib) and flavonoid (quercetin)	Decreases the number of senescent cells and tau aggregation	Phase 1 (NCT04063124 and NCT04785300) Phase 2 (NCT04685590)
Deferiprone	Cellular senescence	Iron-chelating agent	Reduces the amount of reactive oxygen species	Phase 2 (NCT03234686)
AVP-786	Altered intercellular communication	Sigma 1 receptor agonist and NMDA receptor antagonist	Controls neuropsychiatric symptoms	Phase 3 (NCT03393520)
AXS-05	Altered intercellular communication	NMDA receptor antagonist	Controls neuropsychiatric symptoms	Phase 3 (NCT04797715)
Donepezil	Altered intercellular communication	Acetylcholinesterase inhibitor	Improves cognitive impairment	Phase 3 (NCT04661280)
Caffeine	Altered intercellular communication	Adenosine antagonist; non-specific phosphodiesterase inhibitor	Improves cognitive impairment	Phase 3 (NCT04570085)
Blarcamesine (ANAVEX2-73)	Chronic inflammation	M2 autoreceptor antagonist	Improves oxidative stress, protein misfolding, mitochondrial dysfunction and inflammation	Phase 3 (NCT03790709)
Hydralazine	Chronic inflammation	Free radical scavenger	Controls inflammatory response caused by oxidative stress	Phase 3 (NCT04842552)
Icosapent ethyl (IPE)	Chronic inflammation	Purified form of the omega-3 fatty acid EPA	Improves synaptic function and reduces inflammation	Phase 3 (NCT02719327)
NE3107	Chronic inflammation	MAPK-1/3 inhibitor	Reduces activation of proinflammatory NFκB	Phase 3 (NCT04669028)
Canakinumab	Chronic inflammation	Anti-IL-1β monoclonal antibody	Reduces neuroinflammation	Phase 2 (NCT05189106)
Baricitinib	Chronic inflammation	Janus kinase inhibitor	Reduces neuroinflammation	Phase 2 (NCT05189106)
GV-971	Dysbiosis	Algae-derived acidic oligosaccharides	Regulates bacterial imbalance and reduces peripheral and central inflammation	Phase 3 (NCT04520412)

Because cell death is inevitable during ageing, inhibiting the death of various functional cells is also a focus of anti-ageing strategy. Upregulating different anti-apoptotic pathways may serve as an effective strategy for inhibiting ageing. Several anti-apoptotic mechanisms and their essential components have been identified as therapeutic targets for ageing. For example, SIRT6, an NAD^+^-dependent histone deacetylase, plays a vital role in several biochemical processes associated with DNA repair, nutrient metabolism and neurodegeneration, and is a promising therapeutic target for several disorders, including diabetes and ageing. Natural products such as isoquercetin can act as agonists of SIRT6 in the treatment of major human diseases such as AD and can be used to develop selective and potent scaffolds for SIRT6 [305]. Alternatively, targeted removal of senescent cells is considered an important strategy for inhibiting ageing [306]. Senolytics are a class of drugs that can selectively remove senescent cells. The most harmful senescent cells are resistant to apoptosis with upregulation of anti-apoptotic pathways that protect them from autoinflammatory SASPs and allow them to survive while killing neighbouring cells [307]. Senolytics can inactivate these anti-apoptotic pathways, resulting in the apoptosis of the senescent cells through tissue-destructive SASP factors [308]. Tau accumulation has been associated with cellular senescence [10]. In a study, intermittent administration of senolytics via gavage alleviated physical dysfunction and increased survival of old mice [309]. In addition, the combination of senolytics dasatinib and quercetin has demonstrated acceptable safety in the treatment of age-related diseases [310]. However, in-depth investigations are required before senolytics can be safely used in clinical settings.

Additionally, other therapeutic strategies are also under investigations, such as altering intercellular communication, inhibiting chronic inflammation and adjusting dysbiosis (Table 2). Altogether, in-depth research into ageing can help improve the treatment of AD.

## Conclusions and perspectives

Mechanisms underlying ageing and AD are closely related and are a major focus of research at present. Ageing is a multifactorial process that involves the accumulation of molecular and cellular damage over time, resulting in declined biological functions and increased risk of age-related diseases. AD is a progressive and irreversible neurodegenerative disease that primarily affects memory and cognitive function.

Ageing is the most significant risk factor for AD; however, the relationship between ageing and AD, that is, whether ageing is a cause or an effect of AD, remains unclear. Some hallmarks of ageing that contribute to the development and progression of AD have been identified in recent studies [311].

In this review, we systematically summarize the potential molecular mechanisms underlying ageing in AD. AD and ageing accelerate the pathology of each other through a feedback loop. The use of cutting-edge techniques or methods such as MR, multi-omics analysis and single-cell sequencing has enabled researchers to examine the molecular and cellular changes occurring during ageing and AD. Based on the understanding of the mechanisms and shared pathways involved in ageing and AD, novel strategies for preventing and treating age-related diseases such as AD can be developed. Some potential avenues of research include identifying biomarkers of ageing and AD and developing novel drugs such as dasatinib plus quercetin that target cell senescence (NCT04063124 [312], NCT04785300 [18]). In addition, drugs aimed at improving proteostasis and mitochondrial function may also be used for AD treatment.

## Data Availability

Not applicable.

## Abbreviations

Aβ: Amyloid beta
AD: Alzheimer’s disease
CMA: Chaperone-mediated autophagy
CSF: Cerebrospinal fluid
DSB: Double-strand breaks
EV: Extracellular vesicle
GLP-1: Glucagon-like peptide-1
MR: Mendelian randomization
NFT: Neurofibrillary tangles
NSC: Neural stem cells
OPC: Oligodendrocyte progenitor cell
PCDH: Pro-adhesive proteins
ROS: Reactive oxygen species
SASP: Senescence-associated secretory phenotype
SIRT1: Sirtuin-1
TE: Transposable element
TERT: Telomerase reverse transcriptase
UPRmt: Mitochondrial unfolded protein response
